# An Integrated QSAR-MD-DCCM Pipeline: A Predictive Computational Platform for the Rational Design and Dynamic Functional Validation of Dual-Target Directed Ligands

**DOI:** 10.3390/ph19020249

**Published:** 2026-02-01

**Authors:** Shrikant S. Nilewar, Santosh Chobe, Prashik Dudhe, Perli Kranti Kumar, Sandesh Lodha, Akansha D. Raut, Dennys Fernández-Conde, Mohd Farhan, Ghazala Muteeb, Tushar Janardan Pawar

**Affiliations:** 1 Department of Pharmaceutical Chemistry, Maliba Pharmacy College, Uka Tarsadia University, Bardoli 394350, Gujrat, India; shrinilewar@gmail.com (S.S.N.); sandeshlodha@gmail.com (S.L.); 2 Department of Chemistry, M.G.V.’s Loknete Vyankatrao Hiray, Arts, Science and Commerce College, Panchavati, Nashik 422003, Maharashtra, India; chobess222@gmail.com; 3 School of Pharmaceutical Science, Sandip University, Nashik 422213, Maharashtra, India; dudhe.prashik@gmail.com; 4 Department of Pharmaceutical Analysis, JKKN College of Pharmacy, Komarapalayam 638183, Tamil Nadu, India; drpkk1987@gmail.com; 5 Department of Chemistry, School of science, Sandip University, Nashik 422213, Maharashtra, India; rautakansha18@gmail.com; 6 Dirección de Mecatrónica, Universidad Politécnica de Querétaro, Carretera Estatal 420 S/N, El Rosario 76240, Querétaro, Mexico; dennys.fernandez@upq.edu.mx; 7 Department of Chemistry, College of Sciences, King Faisal University, Al-Ahsa 31982, Saudi Arabia; mfarhan@kfu.edu.sa; 8 Department of Nursing, College of Applied Medical Sciences, King Faisal University, Al-Ahsa 31982, Saudi Arabia; 9 Escuela de Ingeniería Química, Universidad Anáhuac Querétaro, Circuito Universidades I, Fracción 2 S/N, Zibatá, El Marqués 76246, Querétaro, Mexico

**Keywords:** machine learning (ML), quantitative structure–activity relationship (QSAR), molecular dynamics (MD), dynamic cross-correlation map (DCCM), Multi-target-directed ligand (MTDL), tubulin, acetylcholinesterase (AChE)

## Abstract

**Background:** The development of Multi-Target-Directed Ligands (MTDLs) has emerged as a significant strategy for addressing complex, overlapping pathologies such as cancer and Alzheimer’s disease (AD). This study aims to provide a robust computational framework for the design of dual-target inhibitors. **Methods:** This study presents an integrated and rigorous computational pipeline combining Quantitative Structure–Activity Relationship (QSAR) modeling, Molecular Docking, and Molecular Dynamics (MD) simulations with Dynamic Cross-Correlation Matrix (DCCM) analysis. Using a dataset of 57 known tubulin inhibitors, two high-performing QSAR models were developed to guide the rational design of 16 novel trimethoxyphenyl-based analogues. **Results:** Following ADMET and drug-likeness filtering, Lead Candidates **15** and **16** were identified. Quantitative activity predictions confirmed their enhanced potency thresholds, which were subsequently validated through static docking against β-tubulin (PDB: 4O2B) and Acetylcholinesterase (PDB: 1EVE). In total, 100 ns MD simulations and MM-GBSA calculations demonstrated superior binding stability and energetically favorable profiles for both targets, while DCCM analysis confirmed the functional synchrony of the protein–ligand complexes. **Conclusions:** The results provide a validated structural hypothesis for dual-target inhibition. The identified leads, **15** and **16**, demonstrate strong predictive potential and are prioritized for chemical synthesis and in vitro biological evaluation.

## 1. Introduction

The pharmaceutical landscape is continually shifting from the “one disease, one target, one drug” paradigm toward sophisticated therapeutic strategies capable of addressing the complex, multifactorial nature of prevalent age-related disorders. This transition has been mandated by the clinical limitations of single-target agents, which may be limited by redundant signaling pathways, compensatory biological mechanisms, and the high failure rate associated with modulating a single node within a dense biological network. Consequently, the concept of Multi-Target-Directed Ligands (MTDLs) has emerged as a significant strategy of modern drug discovery. MTDLs, also known as polypharmacology agents, are single chemical entities designed to simultaneously and beneficially modulate two or more validated targets implicated in a disease cascade. This approach is particularly advantageous for neurodegenerative and oncological diseases, where complex, chronic pathology necessitates intervention at multiple points to achieve clinical efficacy, improve patient adherence, and potentially mitigate drug resistance [1,2,3,4,5].

The strategic importance of multi-target-directed ligands (MTDLs) is increasingly evident in addressing the global health challenges posed by cancer and Alzheimer’s disease (AD). Together, these two pathologies represent a massive public health burden, characterized by high morbidity, soaring socioeconomic costs, and a global patient population that rises exponentially with age [6,7]. While seemingly disparate, accumulating epidemiological evidence points to an intriguing and often paradoxical inverse relationship between cancer and AD incidence, suggesting shared underlying molecular foundations [8,9,10,11,12,13,14,15,16,17,18]. At the cellular level, both conditions converge on dysregulated processes, including oxidative stress, mitochondrial dysfunction, chronic inflammation, and, critically, abnormalities in microtubule (MT) dynamics [19,20,21,22]. While AD pathology is characterized by tau-mediated MT destabilization and neuronal collapse, MT-binding agents targeting the colchicine site, the focus of the current design, similarly promote depolymerization to induce mitotic arrest in rapidly dividing cancer cells.

One of the most compelling common denominators is the MT system. MTs, dynamic polymers of α- and β-tubulin heterodimers, are essential cellular components performing distinct but vital roles in both cancer and neurodegeneration. In cancer cells, MTs are essential for the formation of the mitotic spindle, and inhibitors targeting tubulin polymerization are staples of chemotherapy (vinca alkaloids and taxanes). These agents destabilize or hyper-stabilize MTs, leading to mitotic arrest and apoptosis in rapidly dividing cells [23,24]. In Alzheimer’s disease, however, MTs are central to neuronal architecture, axonal transport, and synaptic integrity. Hyperphosphorylation of the MT-associated protein tau causes MT destabilization, leading to the collapse of the axonal network and the formation of neurofibrillary tangles, which is a hallmark of AD pathophysiology [25,26,27]. Therefore, a single MTDL capable of binding to the tubulin interface, specifically the colchicine binding site on the β-tubulin subunit, offers a promising mechanism: preventing uncontrolled proliferation in cancer and protecting against cytoskeletal collapse and neural degeneration in AD [28,29].

Complementing this structural target, the acetylcholinesterase (AChE) enzyme remains the primary symptomatic target for AD. AChE inhibitors (Donepezil, Rivastigmine, etc.) function by blocking the hydrolysis of the neurotransmitter acetylcholine (ACh) in the synaptic cleft, thereby enhancing cholinergic transmission and temporarily improving cognitive function [30,31]. However, these single-target agents fail to address the underlying disease progression or the MT pathology. The inclusion of an AChE inhibitory domain in a tubulin-targeting scaffold creates a synergistic MTDL: the tubulin-modulating component targets the neurodegenerative etiology, while the AChE-inhibitory component provides necessary symptomatic relief. Crucially, the structural requirements for compounds targeting both the β-tubulin colchicine site and the AChE catalytic/peripheral anionic sites often involve common aromatic and trimethoxyphenyl scaffolds [32,33]. This chemical compatibility reduces the synthetic complexity of creating a potent MTDL, making the dual-target approach both biologically and chemically sound.

The complexity inherent in designing a compound to fit two distinct binding sites and navigate the necessary pharmacokinetic barriers (such as the Blood–Brain Barrier (BBB), crucial for central nervous system targets like AChE) makes traditional high-throughput screening prohibitively costly and time-consuming. This challenge necessitates the application of advanced Computer-Aided Drug Design (CADD) methodologies, an approach central to the field of Computational Chemistry and Chemical Informatics [34,35]. Quantitative Structure–Activity Relationship (QSAR) modeling serves as an important predictive tool, enabling the rapid and cost-effective prediction of biological activity based on key molecular descriptors. QSAR transforms empirical activity data into mathematical models that can rationally guide chemical modifications and filter large virtual libraries before synthesis [36,37]. Following QSAR-guided optimization, molecular docking provides an essential structural validation, predicting the preferred binding pose and affinity (ΔG_bind_) of the MTDL for each target [38,39].

However, static docking predictions often fail to account for the essential dynamic nature of protein–ligand interactions within a physiological environment. Proteins, particularly flexible targets like tubulin and functionally complex enzymes like AChE, undergo significant conformational changes upon ligand binding. This methodological gap is bridged by Molecular Dynamics (MD) simulation, which provides a high-resolution, time-dependent view of complex stability, pose persistence, and the nature of continuous residue contacts (hydrophobic, hydrogen bonding) over hundreds of nanoseconds [40,41]. Furthermore, advanced post-MD analyses, such as the Dynamic Cross-Correlation Map (DCCM), move beyond simple stability checks (Root Mean Square Deviation, RMSD) to uncover essential allosteric or dynamic coupling effects between distant protein domains [42]. Applying this integrated QSAR-MD-DCCM pipeline ensures that predicted MTDL candidates are not only computationally potent but also dynamically stable and structurally valid under simulated physiological conditions, a level of rigor demanded by contemporary chemical informatics research.

**Figure 1:**
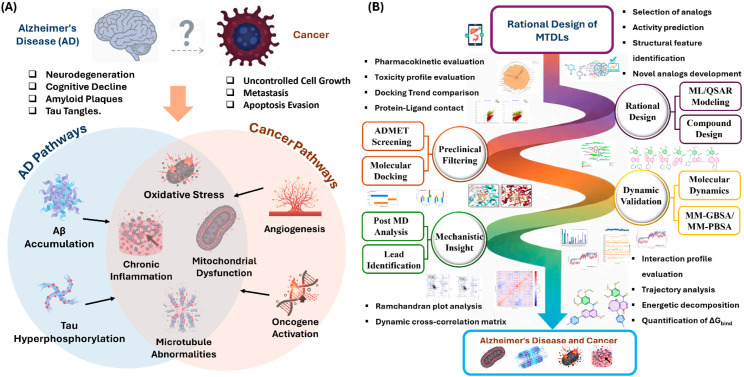
Overview of clinical outcomes and design strategies. (**A**) The divergent clinical outcomes and convergent molecular/cellular hallmarks between Alzheimer’s Disease and Cancer; (**B**) The multi-target directed ligand (MTDL) strategy, including rational design, preclinical filtering, and dynamic validation workflows.

Driven by the need for structurally validated MTDLs against the molecular commonalities of cancer and AD, and leveraging the utility of integrated computational methodologies, the present study introduces a robust QSAR-MD-DCCM workflow for the rational design and virtual validation of novel dual-acting inhibitors. Starting from a focused library of trimethoxyphenyl-containing analogues, we established two predictive QSAR models to guide the rational design of sixteen novel compounds. These compounds were subsequently subjected to extensive in silico ADMET and toxicity profiling to prioritize candidates with optimal drug-like properties and BBB permeability. The most promising candidates were then evaluated against the colchicine binding site of β-tubulin (PDB ID: 4O2B) and the active site of AChE (PDB ID: 1EVE) via molecular docking and rigorous 100 ns MD simulations, and binding free energy calculations (MM-GBSA/MM-PBSA). We conclude by presenting a detailed analysis of ligand stability, binding differentiation, and complex dynamics, using DCCM to provide unprecedented mechanistic insights into the functional allosteric consequences of MTDL binding (Figure 1).

## 2. Results and Discussion

### 2.1. Predictive Validation: QSAR Model Performance

#### 2.1.1. Statistical Validation and Predictive Power

The systematic application of the Genetic Algorithm (GA) coupled with Multiple Linear Regression (MLR) yielded two statistically robust QSAR models, Model 1 and Model 2, aimed at predicting the inhibitory activity (pIC_50_) of trimethoxyphenyl analogs against tubulin polymerization. The performance metrics (Table 1) confirmed that both models adhered strictly to the stringent OECD validation criteria, demonstrating exceptional internal consistency and external predictive capability. To further assess the reliability of the developed QSAR models, the applicability domain (AD) was evaluated using leverage-based statistics in accordance with OECD recommendations. All training and external validation compounds were found to lie within the defined AD boundaries, indicating the absence of influential outliers and confirming that model predictions are interpolation-based rather than extrapolative. Importantly, the newly designed compounds also fall within the descriptor space defined by the training set, supporting the validity of applying the developed QSAR models to guide their rational design and activity prediction.

The high R^2^ values (Model 1: 0.8319; Model 2: 0.8384) demonstrated that over 83% of the variance in the experimental pIC_50_ was explained by the selected descriptors. Critically, the strong cross-validation metrics, including Q^2^ (LOO) (>0.79) and $R_{ext}^{2}$ (>0.85), confirmed the excellent internal predictability and external generalization of both models, respectively, ensuring they are not overfitted to the training data. Furthermore, the Y-randomization test results (Model 1: $R_{Yscr}^{2}$ = 0.0866; Model 2: $R_{Yscr}^{2}$ = 0.0861) demonstrated a clear absence of chance correlation, firmly validating the mathematical significance of the derived equations and underpinning the reliability of the predictive models. To exclude the possibility of chance correlations, Y-scrambling (response permutation testing) was performed after the final descriptor selection and model construction, in accordance with OECD recommendations. This procedure ensures that the randomized models retain the same descriptor combinations while disrupting the response variable, thereby providing a stringent assessment of model robustness. The resulting low R^2^ and Q^2^ values confirmed that the developed QSAR models are not the result of chance correlation.

Model 1 Equation (Topological/Electrostatic Fit):

(1) $${pIC}_{50}=-11.8572+0.9217\cdot {R3s}^{++}+0.9359\cdot map4\_26+34.0115\cdot of0ug+6.4191\cdot ETA\_shape\_p$$

Model 2 Equation (Polarizability/Flexibility):

(2) $${pIC}_{50}=-12.1211+0.2414\cdot Mor15i+0.9746\cdot map4\_26+33.5921\cdot of0ug+6.8459\cdot ETA\_shape\_p$$

#### 2.1.2. Mechanistic Interpretation of Key Descriptors

The efficacy of the QSAR approach, a pillar of this integrated methodology, is strongly validated by the chemical relevance of the selected descriptors, which collectively characterize the optimal molecular features for inhibiting tubulin polymerization at the colchicine binding site (Table S3).

*of0ug* (Molecular Polarizability) emerged as the most dominant determinant, featuring the highest coefficient in both models (Model 1: 34.0115; Model 2: 33.5921). This descriptor quantifies a molecule’s ability to redistribute its electron density, indicating that high electronic polarizability is essential for strong activity. This property facilitates strong van der Waals and induced dipole interactions, which are characteristic of non-covalent binding within the highly aromatic environment of the tubulin colchicine site.

*map4_26* (Topological Complexity), present in both models, represents the topological complexity and molecular architecture. Its positive contribution suggests that a precise degree of molecular complexity and specific branching pattern is required for optimal recognition and fit within the constricted colchicine binding pocket.

*ETA_shape_p* (3D Conformational Flexibility), also highly weighted in both models (coefficient around 6.5), quantifies the responsiveness of the molecule’s three-dimensional shape to conformational changes. The strong positive correlation confirms that a certain level of molecular flexibility and shape adaptability is advantageous, enabling the ligand to optimally adjust its conformation to the dynamic structural changes that occur in β-tubulin during polymerization inhibition.

The two models are further differentiated by unique descriptors: *R3s++* (Steric Accessibility) in Model 1 emphasizes that reduced steric hindrance is a prerequisite for effective binding within the spatially confined pocket. Conversely, *Mor15i* (Electronic Density) in Model 2, a 3D-MoRSE descriptor, reinforces the need for electronic complementarity and specific mass distribution to fine-tune the inhibitory potential.

Given its slightly superior R^2^ and the inclusion of descriptors emphasizing both electronic density (*Mor15i*) and flexibility, Model 2 was prioritized as the primary design tool. Model 1 served as an essential cross-validation tool focused on steric fit, ensuring that the subsequently designed analogs were simultaneously robust across key electronic and spatial parameters, a dual requirement that critically informed the rational structural modifications detailed next.

The structural relevance of these descriptors is clearly reflected across the 57-compound training set (Table S2, Figure S3). For instance, molecules with high molecular polarizability (*of0ug*), such as the sulfur-bridged analogues (compounds **20–25**), consistently displayed higher pIC_50_ values, confirming that electronic redistribution is a primary driver for potency in this chemical space. Similarly, the importance of topological complexity (*map4_26*) is evident in the superior activity of structures with specific branching patterns on the indole or phenyl rings, such as the ethoxy-substituted derivative (compound **30**). Conversely, the steric accessibility descriptor (*R3s++*) in Model 1 correctly identified that excessive bulk at the trimethoxyphenyl interface reduces inhibitory potential, as observed in the lower activity of the more sterically hindered derivatives in the dataset.

It should be noted that the absolute values of the regression coefficients were not used as a direct quantitative measure of descriptor importance, as the descriptors differ in scale and numerical range. Instead, descriptor relevance was evaluated based on their statistical significance, consistency across both models, and chemical interpretability in relation to known tubulin–ligand interactions. This qualitative, mechanistic interpretation avoids bias arising from descriptor scaling effects.

### 2.2. Design and Preclinical Filtering: Novel Analogs and ADMET Analysis

#### 2.2.1. QSAR-Guided Rational Design

Leveraging the mechanistic insights generated by the predictive QSAR models, particularly the high importance placed on molecular polarizability (*of0ug*), topological complexity (*map4_26*), and conformational flexibility (*ETA_shape_p*), a panel of 16 novel compounds was rationally designed around the trimethoxyphenyl core. The primary objective was to systematically perturb these key descriptors to optimize the predicted pIC_50_ and enhance the compound’s dual-targeting profile. Structural modifications included the addition of polar groups (amino, benzoic acid), substitution of heteroatoms (chlorine, sulfur), and the incorporation of fused aromatic rings (naphthalene, pyridone-fused systems). These changes were targeted to refine lipophilicity, rigidity, and the capacity for π-stacking interactions essential for both tubulin and AChE binding sites. The specific design rationale for each compound (Table 2, Figure S5) systematically links the chemical modification to the targeted descriptor perturbation. All newly conceived structures were optimized using the MMFF94 force field in MarvinSketch to obtain energetically favorable 3D coordinates, which served as the consistent input for all subsequent in silico validation steps.

**Figure 2:**
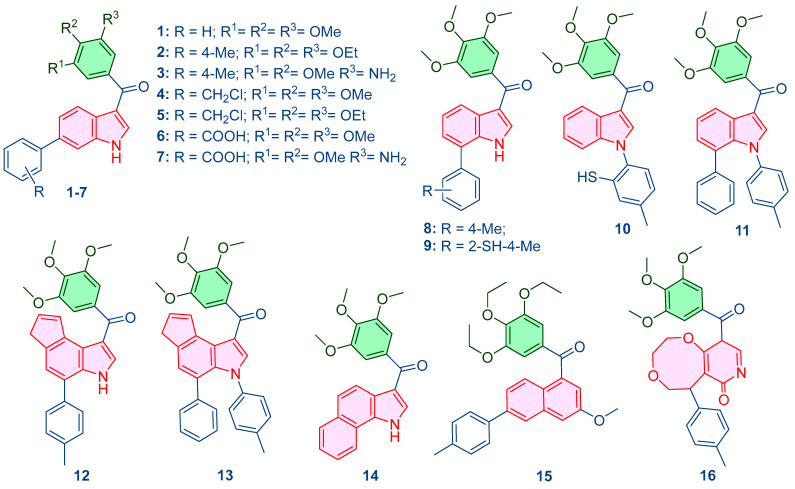
Designed compounds using the generated QSAR model.

To quantitatively validate the rational design, the pIC_50_ values for all 16 novel analogues were predicted using both developed QSAR models (Table 2, Figure 2). The results confirm that the targeted structural perturbations successfully translated into the intended potency gains, with lead candidates **15** and **16** exhibiting high predicted inhibitory activity, thereby justifying their selection for advanced dynamic validation.

#### 2.2.2. ADMET Profile and Lead Candidate Selection

Following the rational design, all 16 novel compounds underwent comprehensive ADMET and toxicity screening using ADMETlab 3.0 and ProTox-II platforms to ensure their preclinical viability (Table S4). This pre-screening filter was critical for identifying candidates with favorable pharmacokinetic profiles before proceeding to resource-intensive structural simulations. Given the dual requirement for systemic efficacy (cancer) and CNS penetration (AChE targeting), selection criteria prioritized candidates with high BBB permeability, favorable lipophilicity (logP ideally 2–5) for good oral bioavailability, and minimal predicted safety liabilities.

This systematic, multi-parameter evaluation led to the identification of Compounds **15** and **16** as the optimal lead candidates (Table 3). Compound **15** displayed favorable lipophilicity (log P of 2.417, log D of 2.718) and a predicted BBB value of 0.564, indicating high bioavailability and the potential to cross the BBB for efficient AChE targeting. Its ProTox analysis suggested a low probability of key toxicities. Compound **16** showed optimized lipophilicity (log P of 3.229, log D of 3.073) and an exceptionally high BBB penetration value (0.998), suggesting highly effective CNS targeting. While it exhibited a safe profile for neurotoxicity and cardiotoxicity, its elevated hepatotoxicity score and CYP2E1 interaction were noted as areas requiring future experimental investigation. This observation should therefore be interpreted with caution, as in silico hepatotoxicity flags often reflect potential metabolic liabilities linked to increased lipophilicity and hepatic metabolism rather than direct evidence of intrinsic toxicity. The predicted involvement of CYP2E1 instead highlights a plausible metabolic clearance pathway, suggesting that targeted structural refinement or metabolic hotspot modification could be explored in future optimization efforts to reduce possible liver-related risks. Together, Compounds **15** and **16** exhibited favorable but distinct pharmacokinetic characteristics that support their evaluation as complementary dual-target candidates. Specifically, Compound **15** showed a more balanced lipophilicity and safety profile, which is advantageous for systemic exposure and AChE targeting, whereas Compound **16** displayed higher lipophilicity and exceptional predicted BBB penetration, favoring CNS access but with identified metabolic liabilities. These differences justify their advancement as complementary leads for further structural and dynamic validation rather than as interchangeable candidates.

The high-confidence virtual validation of leads **15** and **16** provides a robust structural foundation for immediate chemical synthesis and in vitro assessment. While these computational predictions significantly de-risk the discovery process, empirical assays remain the definitive step for translating these MTDL candidates into clinical leads. In this regard, early in vitro evaluation, including hepatotoxicity screening and metabolic stability assays, will be important to experimentally clarify and further refine the safety profile of Compound 16 during subsequent lead optimization.

### 2.3. Structural Validation: Dual-Target Molecular Docking and Affinity Assessment

**Figure 3:**
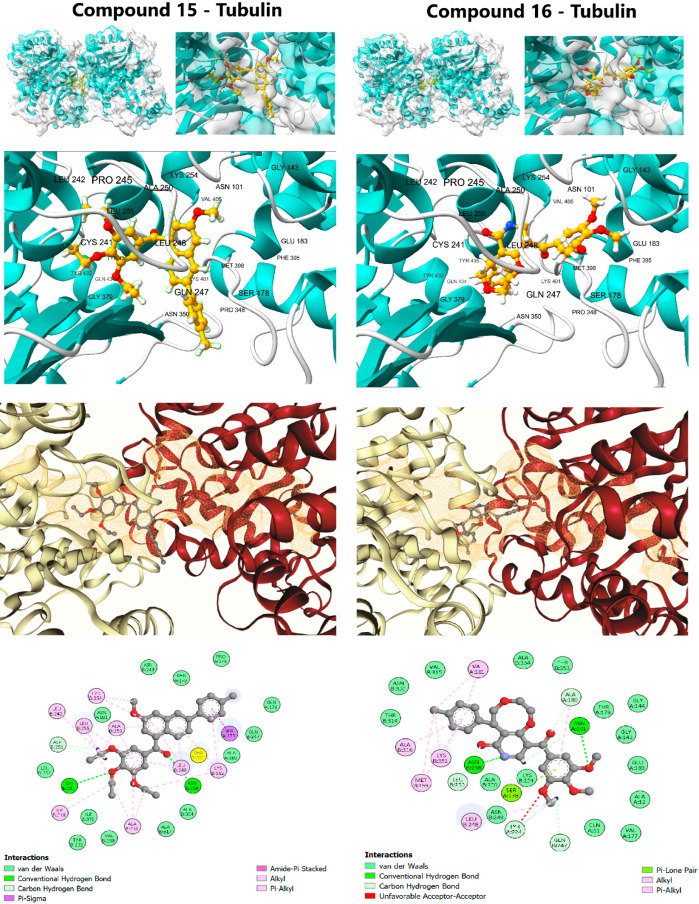
Detailed molecular docking interactions of Lead Candidates **15** and **16** within the colchicine binding site of β-tubulin (PDB ID: 4O2B).

**Figure 4:**
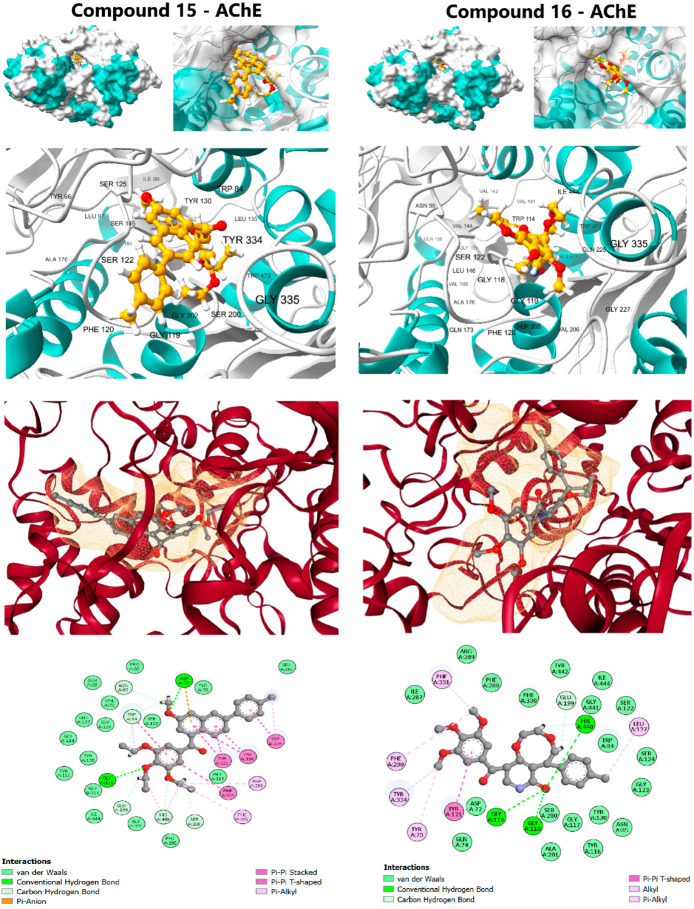
Molecular docking interactions of Lead Candidates **15** and **16** against the active site of Acetylcholinesterase (AChE) (PDB ID: 1EVE).

The initial structural validation of the rigorous computational workflow was performed by assessing the static binding affinity (ΔG) and detailed interaction profiles of the QSAR-filtered lead candidates, compounds **15** and **16**, against their respective targets. For the anticancer target, the colchicine-binding site of Tubulin (PDB ID: 4O2B) was used, and for the anti-Alzheimer’s target, the active site of AChE (PDB ID: 1EVE) was employed. The accuracy of the docking protocol was rigorously validated by successfully reproducing the crystallographic poses of the reference ligands, Colchicine (RMSD: 0.172 Å) and Donepezil (RMSD: 0.532 Å), confirming the geometric accuracy of the method (Figure 3 and Figure 4, Tables S5 and S6).

The calculated binding free energies (Table 4) revealed target-specific binding characteristics, strongly supporting the feasibility of the dual-targeting strategy. Compound **16** showed a marginally superior predicted affinity for the Tubulin binding site (ΔG: −10.0 kcal/mol), making it the prioritized anticancer candidate, while compound **15** exhibited superior predicted affinity for the AChE active site (ΔG: −10.6 kcal/mol), confirming its strong potential as an anti-Alzheimer’s agent.

#### 2.3.1. Tubulin Binding Profile (Anticancer Target)

The affinity of compound **16** (ΔG: −10.0 kcal/mol) was nearly identical to the positive control, Colchicine (ΔG: −10.1 kcal/mol), suggesting highly effective molecular recognition at the interface of the α- and β-tubulin subunits. Compound **16** achieved this potent binding via an extensive interaction profile: Hydrogen Bonding with key residues Gln11, Asn101, and Ala180 (Chain A) and Asn258 (Chain B); and π-Alkyl Contacts with hydrophobic residues Leu248, Ala250, Lys254, and Leu255 (Chain B) [43]. This widespread engagement, spanning both chains, explains its superior affinity and strong propensity to destabilize the MT structure. Compound **15** (ΔG: −9.7 kcal/mol) displayed a similar but less extensive profile, relying primarily on π-Alkyl interactions (Chain B) and a single hydrogen bond to Asn258. Although classical colchicine-site ligands primarily interact with β-tubulin, the binding pocket lies at the α/β interface, and larger or more flexible ligands may extend across this region depending on their orientation and conformational adaptability. In this case, the scaffold size and flexibility of compounds **15** and **16** likely allow transient contacts with residues from both subunits in the docking poses.

Importantly, such docking-derived interaction patterns do not directly predict functional effects on microtubule dynamics, as they represent static binding geometries. MD simulations were therefore employed to evaluate whether these interfacial contacts persist over time or relax toward a more localized binding mode, providing a more realistic assessment of complex behavior.

#### 2.3.2. Acetylcholinesterase Binding Profile (Anti-Alzheimer’s Target)

Compound **15** demonstrated a strong affinity (ΔG of −10.6 kcal/mol), approaching the clinical standard Donepezil (ΔG: −11.8 kcal/mol) and confirming its potential for cholinesterase inhibition. Its interaction profile was diverse and indicative of a dual-binding site mechanism: Hydrophobic Interactions, including π-Alkyl contacts at Phe290 and Phe331; π-π Stacking interactions with aromatic residues, notably Tyr121, Trp279, Phe330, and Tyr334; and Hydrogen Bonding with Asp72 and Gly118. The trimethoxyphenyl scaffold effectively engaged the peripheral anionic site (PAS) residues, while the rest of the molecule penetrated deeper into the active site Gorge. Compound **16** (ΔG: −9.7 kcal/mol) also formed favorable contacts but lacked the extensive π-π stacking seen in compound **15**, resulting in a diminished affinity. The complementary target affinity profiles observed in the docking study were used to guide the subsequent rigorous dynamic validation step.

### 2.4. Dynamic Validation: 100 ns MD Simulation

#### MD Simulation and Stability Analysis

To move beyond static binding predictions and perform a rigorous validation of the integrated workflow, 100 ns all-atom MD simulations were conducted for the four final complexes, demonstrating the dynamic behavior of the lead compounds under simulated physiological conditions. This process served as a high-resolution filter, confirming the stability and permanence of the strong binding poses observed in the docking study.

**Figure 5:**
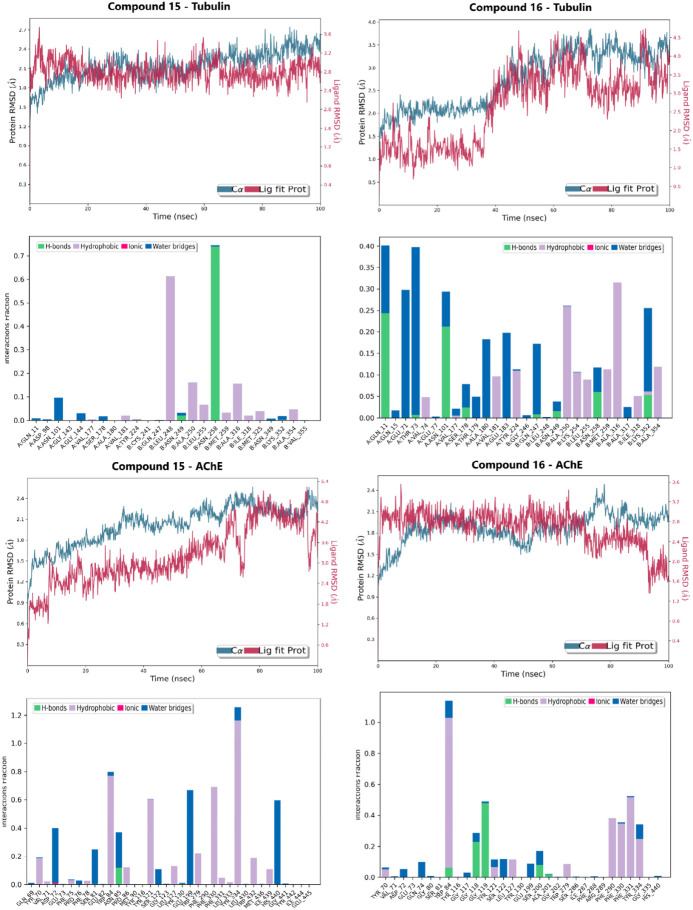
Molecular dynamics (MD) simulation results for Lead Candidates **15** and **16** over a 100 ns trajectory. The panels display the Protein/Ligand RMSD and the interaction fractions for both Tubulin and AChE complexes.

The RMSD relative to the protein served as the primary metric for tracking conformational stability (Figure 5 and Figure S6). The compound **16**-Tubulin complex exhibited superior stability, maintaining RMSD values largely within the 1.0–4.0 Å range, with the main body of the compound remaining fixed inside the colchicine binding site for the entire simulation. The Compound **15**-Tubulin complex also proved highly rigid, staying well below 2.0 Å RMSD for most of the trajectory. Conversely, the AChE complexes showed slightly higher, yet constrained, fluctuations: Compound **15**-AChE fluctuated between 1.8–4.8 Å, settling into a stable secondary conformation after an initial ring rotation, while compound **16**-AChE remained rigid, oscillating between 1.6–3.2 Å.

The RMSF of the ligands further differentiated the binding dynamics, indicating that the compounds utilize complementary stabilization mechanisms. Compound 15 displayed high flexibility in the AChE site (RMSF: 3.0 Å), suggesting adaptation to engage multiple hydrophobic residues and continuous H-bonding, but low flexibility in the Tubulin site (RMSF: 1.5 Å). Compound **16** showed the reverse trend: low flexibility in the AChE pocket (RMSF: 1.5 Å) but higher flexibility in the Tubulin pocket (RMSF: 3.0 Å), indicating the motion required to react to the fluctuating tubulin structure and interact with multiple residues.

### 2.5. Energetic Validation: MM-GBSA/MM-PBSA Analysis

The MM-GBSA/MM-PBSA decomposition of the MD trajectories provided valuable insights into the energetic basis of binding for compounds **15** and **16** with both tubulin and AChE, supporting their potential as dual-target anticancer and anti-Alzheimer’s agents. In all four complex systems, the gas-phase contributions were markedly favorable and were dominated by van der Waals and electrostatic factors. This highlighted the importance of tight packing and aromatic stacking with residues embedded within both the colchicine site of tubulin and the aromatic active site region of AChE. The consistently stabilizing van der Waals profiles reflected the contribution of the tri-methoxy substituted phenyl core, enabling the extensive hydrophobic complementarity. The polar solvation penalties observed are typical for the positioning of the ligand within deep hydrophobic binding sites.

The MM-GBSA and MM-PBSA approaches yielded quantitatively different binding free energy values, which is not unexpected given their distinct treatments of polar solvation effects. In particular, MM-PBSA can produce less favorable or even positive total energies for flexible ligands or interfacial binding sites; therefore, the results were interpreted in a comparative rather than absolute manner. The conformational entropy contribution was not explicitly included, as its reliable estimation is computationally demanding and commonly omitted in comparative studies. Standard dielectric settings were employed, using an internal dielectric constant of 1 and a solvent dielectric constant of 80 to represent bulk aqueous conditions consistently across all systems.

**Figure 6:**
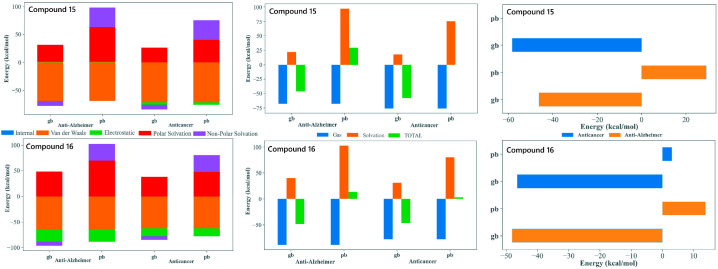
MM-GBSA/MM-PBSA analysis of binding free energy (ΔG_bind_) for compounds **15** and **16**. The analysis compares the energetic contributions (van der Waals, Electrostatic, Solvation) to the stable binding of the lead candidates against the Anti-Alzheimer (AChE) and Anticancer (Tubulin) targets. Favorable ΔG_bind_ values across both MM-GBSA (gb) and MM-PBSA (pb) methods support their dual-inhibitory potential.

Both compounds display comparably favorable total binding energies across tubulin and AChE, supporting their suitability as dual-acting candidates. Compound **15**, with an extended biphenyl substituent, achieved stable binding through enhanced π-stacking and hydrophobic enclosure. This is particularly advantageous for tubulin interactions but also remains beneficial in AChE. Compound **16**, with a heterocyclic imide-linked fused ring system, showed a good balance between electrostatic interactions and solvation effects. This behavior works together with the hydrophobic contribution of its trimethoxyphenyl group, allowing it to interact favorably within both binding pockets. This difference in structural features between the two ligands helps explain why both maintained favorable binding energies across the two targets despite their varying energetic contributions. Overall, the MM-GBSA/MM-PBSA results supported the selection of **15** and **16** as strong candidates for dual inhibition. Both compounds demonstrated the ability to bind effectively to tubulin as well as AChE. They used slightly different but complementary interaction patterns. Their binding energies remained stable over the full 100 ns MD simulations. Their structural flexibility allowed them to adapt well within two very different binding environments (Figure 6).

### 2.6. Functional Validation: Post-MD Mechanistic Insights

The final methodological steps involved performing functional analysis on the 100 ns trajectories, serving as the ultimate verification point of the predictive workflow.

#### 2.6.1. The Ramachandran Plot Analysis

**Figure 7:**
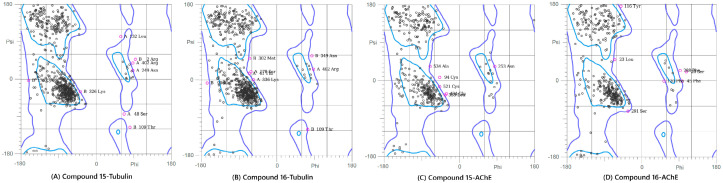
Ramachandran Plot analysis of the modeled protein structures after the 100 ns Molecular Dynamics simulations. The plots show the backbone dihedral angle distribution of residues, confirming the stereochemical quality of the final ligand-bound complexes: (**A**) Compound **15**-Tubulin, (**B**) Compound **16**-Tubulin, (**C**) Compound **15**-AChE, and (**D**) Compound **16**-AChE. The high percentage of residues in the favored and allowed regions validates the conformational suitability of all four systems for further analysis.

This analysis was performed on the final (100 ns) snapshot of each molecular dynamics trajectory using the MolProbity server to rigorously validate the stereochemical quality and conformational integrity of the target proteins post-ligand binding (Figure 7 and Figure S7). The results consistently demonstrated that the ligand-induced conformations for all four complexes were structurally sound and highly reliable. Specifically, the complexes showed high percentages of residues in the favored regions (ranging from 86.5% to 91.2%) and allowed regions (ranging from 96.8% to 99.1%). A quality threshold of >85% of residues in the favored regions is considered excellent for post-MD analyses, and the low number of outliers, confined primarily to flexible loop regions, unequivocally confirmed the stereochemical validity of the models. The Ramachandran plot analysis was included as a post-simulation stereochemical quality check to confirm that protein backbone conformations remained within physically allowed regions following MD simulations. Although this analysis does not provide direct insight into binding affinity or dynamic behavior, it is a standard validation step used to ensure that no unrealistic backbone distortions arise during prolonged simulations. The high proportion of residues in favored and allowed regions therefore supports the structural integrity of the simulated complexes and the reliability of subsequent post-MD analyses.

#### 2.6.2. Dynamic Cross-Correlation Matrix (DCCM) Analysis

**Figure 8:**
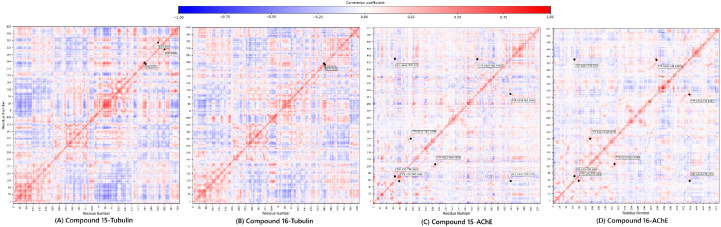
Dynamic cross-correlation matrix of compounds **15** and **16**.

The final, high-value component of the integrated pipeline was the DCCM Analysis. This analysis quantifies the concerted motions between residue pairs, providing a functional, allosteric context for the observed stability. The DCCM maps (Figure 8) confirmed the compounds’ complementary dynamic signatures.

For Tubulin, compound **15** exhibited a complex pattern of both positive and negative correlations, suggesting it engages in broader, more widespread structural adjustments across the α/β interface (greater structural flexibility). Conversely, compound **16** showed a more simplified and localized correlation profile, aligning with its rigid, focused binding mode.

For AChE, both complexes maintained positive correlations in critical pairs (Tyr70-Trp84, Tyr121-Glu199, and Tyr334-His440) [44]. Crucially, compound **15** showed a distinct negative correlation between Gly441-Tyr70, a key pair regulating the gorge entrance. The presence of this anti-correlation in **15** suggests it is more effective at modulating the dynamic opening/closing of the AChE gorge, reinforcing its superior predicted inhibitory affinity and deeper mechanistic engagement observed in the docking study.

The DCCM analysis serves as the functional validation of the entire predictive workflow, reinforcing that compound **15** employs a broader, more flexible engagement, while compound **16** uses a more focused, rigid strategy, suggesting the potential and structural viability of the lead candidates.

## 3. Materials and Methods

### 3.1. Dataset Preparation and QSAR Modeling Rationale

The development of robust QSAR models commenced with an exhaustive literature search utilizing the ChEMBL and BindingDB databases to create a high-quality dataset of 57 molecules (Table S3). The key inclusion criterion was the presence of the trimethoxyphenyl scaffold, a pharmacophore known for its affinity to the colchicine binding site on tubulin and documented tubulin polymerization inhibitory activity (IC_50_). Biological activity values (IC_50_) were uniformly converted to the negative logarithmic scale (pIC_50_) to ensure a normal distribution and reduce data skewness, which is essential for stable Multiple Linear Regression (MLR) modeling. The final dataset of 57 compounds was systematically divided to ensure rigorous validation: 10 compounds (≈17.5%) were designated as the external validation set to assess predictive capability on unseen data, while the remaining 47 compounds formed the training set for model generation and internal cross-validation. This division strategy maximized the training set size, ensuring model stability and adequate descriptor space coverage while maintaining substantial external validation power, adhering strictly to OECD principles for QSAR development [36,45].

### 3.2. Descriptor Generation & Pre-Filtering

Molecular structures for all 57 compounds (Figure S3) were rigorously optimized using the MMFF94 force field within MarvinSketch to ensure consistent, low-energy three-dimensional conformations. Subsequently, a massive initial pool of 11,829 molecular descriptors was generated using specialized software packages, including PaDEL-Descriptor, alvaDesc, MORDRED, and MERA via the OCHEM server [46]. This extensive descriptor space, encompassing constitutional, topological, geometrical, electronic, and hybrid properties, was intentionally broad to provide the Genetic Algorithm (GA) with the widest possible feature set for robust structure–activity relationship identification. To mitigate the inherent risks of data overfitting and multicollinearity associated with such large descriptor pools, a systematic two-stage pre-filtering procedure was implemented. First, descriptors exhibiting the same value across more than 80% of the dataset were removed; second, a threshold of 95% correlation was applied to eliminate redundant descriptor pairs. This methodical pre-filtering eliminated 7943 descriptors, resulting in a refined and highly informative set of 3886 descriptors for the subsequent QSAR model construction phase.

The QSAR model construction utilized a GA primarily as a feature selection technique to navigate the high-dimensional descriptor space (>3800 variables) and mitigate the risk of over-parameterization. The GA identified optimal subsets of descriptors by maximizing a fitness function based on the Quik Rule (Q), which ensures low inter-correlation between descriptors and high model parsimony and the Leave-One-Out (LOO) cross-validation (Q^2^). Once the ideal descriptor combinations were evolved, Multiple Linear Regression (MLR) was employed to calculate the final regression coefficients and establish the predictive equations. Prior to model development, descriptor selection was guided by genetic algorithm optimization rather than coefficient magnitude alone. No descriptor weighting or ranking was inferred solely from raw regression coefficients, and descriptor relevance was interpreted in a comparative and mechanistic manner across models.

### 3.3. Molecular Sketching and Geometry Optimization

Leveraging the mechanistic insights derived from the QSAR models, a series of 16 novel compounds was rationally designed based on the original trimethoxyphenyl scaffold. The design strategy was guided by the positive contribution of the identified key descriptors. For instance, structural modifications such as the addition of chlorine or ethoxy groups were introduced to modulate *Mor15i* (electronic density) and *map4_26* (topological complexity), while the introduction of fused rings or changes in substituent positions were aimed at optimizing *ETA_shape_p* (conformational flexibility) and steric fit (*R3s++*). Specific modifications included altering the aromaticity, introducing flexible linkers, and changing the polarity of key functional groups to maximize the predicted pIC_50_ for tubulin inhibition. All newly sketched structures were subsequently subjected to geometry optimization using the MMFF94 force field in MarvinSketch to obtain energetically favorable 3D coordinates, which served as the input for all subsequent ADMET and docking calculations [32,47,48,49].

### 3.4. ADMET Prediction and Selection of Lead Candidates

Following the rational QSAR-guided design, all 16 novel compounds were subjected to comprehensive ADMET profiling to rigorously filter candidates before proceeding to resource-intensive structural simulations. This critical in silico screen employed the ADMETlab 3.0 and ProTox-II platforms to predict essential pharmacokinetic parameters, including Caco-2 permeability, BBB penetration, aqueous solubility (logS), lipophilicity (logP and logD), metabolism via CYP isoforms, and general toxicity risks such as hepatotoxicity and mutagenicity. Given the dual-target nature of the study, requiring systemic efficacy for cancer and CNS penetration for Alzheimer’s Disease, the selection criteria were stringent, prioritizing candidates with high BBB permeability (essential for AChE targeting), favorable logP (ideally 2–5) for oral bioavailability, and minimal predicted safety liabilities. This systematic, multi-parameter evaluation led to the identification of Compounds **15** and **16** as the optimal lead candidates (Figure S4). Compound **15** was selected for its balanced physicochemical profile and predicted BBB permeability, while Compound **16** was advanced due to its exceptional BBB penetration prediction, justifying their prioritization for the subsequent molecular docking and MD simulations to validate their structural binding hypotheses [50].

### 3.5. Molecular Docking & Validation

The dual-target potential of the two selected leads, Compounds **15** and **16**, was structurally evaluated using molecular docking simulations against their respective targets. For the anticancer target, the crystal structure of β-tubulin in complex with colchicine (PDB ID: 4O2B) was used [43]. For the anti-Alzheimer’s target, the crystal structure of AChE (PDB ID: 1EVE) was utilized [44]. The proteins were pre-processed (removing water molecules, standardizing protonation states) using the Discovery Studio Visualizer and SwissPDB Viewer. Docking calculations were performed using AutoDock Vina (version 1.2.0) integrated within the CB-Dock2 server, which automatically defines the optimal binding site cavity, ensuring an unbiased “blind docking” approach [38,51]. Validation of the docking methodology was performed by redocking the co-crystallized ligands (Colchicine for 4O2B and Donepezil for 1EVE). The method was accepted only after the Root Mean Square Deviation (RMSD) between the docked and crystal poses was less than 2.0 Å [52]. The final predicted binding affinities (ΔG in kcal/mol) and the nature of the specific protein-ligand interactions (H-bonds, π-stacking, hydrophobic contacts) were meticulously analyzed using Discovery Studio Visualizer and UCSF Chimera for both compounds against both targets [53].

### 3.6. MD Simulation and Post-MD Analysis

To comprehensively assess the stability, flexibility, and permanence of the protein-ligand interactions observed in the static docking poses, 100 ns all-atom MD simulations were conducted for the four final complexes: **15**-Tubulin, **16**-Tubulin, **15**-AChE, and **16**-AChE. The simulations were performed using the Desmond module within the Schrödinger Suite. Each system was prepared by placing the docked complex in a periodic boundary condition box solvated with TIP3P water molecules and neutralized with Na^+^ and Cl^-^ ions. The OPLS3e force field was used to describe all atomic interactions. After a thorough minimization and pre-equilibration phase, the production run was executed for 100 ns under constant temperature (300 K) and pressure (1 bar) conditions, with trajectories saved every 50 ps [54].

Post-MD Analysis was critical for interpreting the dynamic stability and functional integrity: trajectory analysis (Protein/Ligand RMSD and residue RMSF) monitored stability and flexibility; interaction timelines tracked the persistence of specific residue contacts; the Ramachandran Plot validation ensured the stereochemical quality and conformational suitability of the final complex structures using the MolProbity server [55]; and finally, the advanced Dynamic Cross-Correlation Matrix (DCCM) analysis, performed with the MD-TASK suite, was used to quantify the functional connectivity and allosteric communication within the protein induced by ligand binding [56].

To provide the energetic basis for the observed dynamic stability, the binding free energy (ΔG_bind_) for the four protein–ligand complexes (**15**-Tubulin, **16**-Tubulin, **15**-AChE, and **16**-AChE) was calculated using the MM-GBSA and MM-PBSA methodologies. This was performed on the MD trajectories using the Prime module of the Schrödinger Suite. The analysis was conducted on the final 50 ns (500 frames) of the 100 ns production run to ensure the inclusion of only equilibrated conformations. The free energy of binding was calculated according to the general equation:

$$∆G_{bind}=∆E_{MM}+∆G_{solv}-T∆S$$

where ΔE_MM_ represents the change in molecular mechanics energy (sum of ΔE_vdw_ and ΔE_elec_), ΔG_solv_ represents the change in solvation energy (polar and non-polar components), and TΔS is the change in conformational entropy. The non-polar contribution to solvation energy was calculated using the Solvent Accessible Surface Area (SASA) model. The entropy term (TΔS) was neglected due to its computational intensity, which is common practice for comparative studies. The focus was on the most favorable non-covalent terms: van der Waals and electrostatic energies, as they dominate the gas-phase energy in these systems.

## 4. Conclusions

This study successfully established an integrated QSAR-MD-DCCM pipeline for the rational discovery and dynamic validation of MTDLs, satisfying all stringent OECD criteria. Two highly accurate, statistically robust QSAR models (R^2^ > 0.83) were developed from a focused trimethoxyphenyl scaffold library, where mechanistic interpretation demonstrated that molecular polarizability (*of0ug*), topological complexity (*map4_26*), and conformational flexibility (*ETA_shape_p*) are the indispensable key determinants for effective tubulin polymerization inhibition. These quantitative insights directly guided the rational design of 16 novel analogues, demonstrating the pipeline’s utility in early-stage Machine Learning application. The leads identified through this initial filtering, compounds **15** and **16**, were selected based on optimized drug-likeness and their high predicted capacity for BBB penetration, an essential feature for effective AChE targeting. Molecular docking and rigorous 100 ns MD simulations confirmed dynamically stable and persistent binding for both leads against both Tubulin (PDB ID: 4O2B) and AChE (PDB ID: 1EVE). Furthermore, MM-GBSA/MM-PBSA binding free energy calculations provided essential energetic validation, showing highly favorable total binding energies (ΔG_bind_) that primarily stemmed from stabilizing van der Waals interactions. This analysis revealed a complementary, target-differentiated potency: Compound **16** showed marginally superior predicted affinity for the Tubulin binding site (ΔG: −10.0 kcal/mol), while compound **15** exhibited superior predicted affinity for the AChE active site (ΔG: −10.6 kcal/mol). Post-MD Ramachandran analysis confirmed the high stereochemical integrity of all four protein–ligand complexes. Furthermore, the DCCM analysis provided the final and highest level of functional validation, revealing complementary dynamic signatures: Compound **15** was found to induce a broader range of dynamic interactions and allosteric coupling, suggesting a more flexible engagement, while compound **16** exhibited a more focused and rigid inhibition strategy. In conclusion, this rigorous, integrated QSAR-MD-DCCM pipeline represents a highly predictive and robust platform for the de novo design and dynamic validation of multi-target therapeutics. The successful virtual validation of two distinct dual-acting candidates (**15** and **16**) serves as compelling proof-of-concept for this advanced computational workflow, ready for immediate experimental synthesis and *in vitro* validation.

## Figures and Tables

**Table 1:** Model validation parameters.

Metric	Model 1	Model 2	OECD Threshold
$R_{Yscr}^{2}$	0.0866	0.0861	<0.20
$Q_{Yscr}^{2}$	−0.1499	−0.1512	<0.20
R^2^ (Training)	0.8319	0.8384	>0.60
Adjusted R^2^	0.8159	0.8230	Close to R^2^
Q^2^ (LOO Cross-validation)	0.7940	0.7976	>0.50
Q^2^ (LMO Cross-validation)	0.7804	0.7976	>0.60
$R_{ext}^{2}$ (External Prediction Set)	0.8565	0.8633	>0.60
CCC (External)	0.9197	0.9252	>0.85
RMSE (Training)	0.1360	0.1333	Lower is better
MAE (training)	0.1067	0.1082	Lower is better
r^2^m (average)	0.7926	0.8022	>0.50
Δr^2^m	0.0340	0.0097	<0.20

**Table 2:** Rational design of novel MTDL analogues and quantitative activity prediction using the developed QSAR models.

Comp. No.	Targeted Descriptors	Design Rationale	pIC_50_	
			Equation (1)	Equation (2)
1	*Mor15i*, *map4_26*, *ETA_shape_p*, *R3s++*	Phenyl addition to 6th position enhances shape and aromaticity for optimized binding.	10.868	5.251
2	*Mor15i*, *map4_26*, *of0ug*, *ETA_shape_p*, R3s++	Ethoxy substitution modifies lipophilicity and molecular geometry for better receptor interaction.	10.782	4.399
3	*Mor15i*, *map4_26*, *ETA_shape_p*, *R3s++*	Methoxy-to-amino replacement alters polarity and shape for improved receptor targeting.	10.311	4.377
4	*Mor15i*, *map4_26*, *of0ug*, *ETA_shape_p*, *R3s++*	Chlorine addition alters electron density and lipophilicity, impacting binding and molecular shape.	10.018	4.317
5	*Mor15i*, *map4_26*, *of0ug*, *ETA_shape_p*, *R3s++*	Chlorine and ethoxy substitution affect hydrophobicity, shape, and receptor fit.	10.663	4.894
6	*Mor15i*, *map4_26*, *ETA_shape_p*, *R3s++*	Benzoic acid replaces Tolyl, increasing aromaticity and modifying shape for better binding.	11.213	5.053
7	*Mor15i*, *map4_26*, *of0ug*, *ETA_shape_p*, *R3s++*	Amino substitution and benzoic acid replace Tolyl, enhancing hydrophilicity, shape, and binding.	11.506	4.751
8	*Mor15i*, *map4_26*, *ETA_shape_p*, *R3s++*	Tolyl group affects topological and spatial properties for optimized binding.	10.144	4.494
9	*Mor15i*, *map4_26*, *ETA_shape_p*, *R3s++*	Tolyl and SH substitution increases aromaticity, modifying binding interactions.	10.778	5.065
10	*Mor15i*, *map4_26*, *ETA_shape_p*, *R3s++*	Tolyl at 1st position with SH alters shape and polarity for improved receptor fit.	10.423	4.395
11	*Mor15i*, *map4_26*, *ETA_shape_p*, *R3s++*	Tolyl at 1st position and phenyl at 7th position modifies geometry and receptor interactions.	9.633	4.082
12	*Mor15i*, *map4_26*, *ETA_shape_p*, *R3s++*	Tolyl at 7th position and fused 5-membered ring enhances aromaticity and binding.	10.073	4.027
13	*Mor15i*, *map4_26*, *ETA_shape_p*, *R3s++*	Tolyl at 1st position, phenyl at 7th, and fused 5-membered ring improve aromaticity and binding.	10.649	5.020
14	*Mor15i*, *map4_26*, *ETA_shape_p*, *R3s++*	Fused 6-membered ring increases aromaticity and rigidity for better receptor interaction.	10.627	5.061
15	*Mor15i*, *map4_26*, *ETA_shape_p*, *R3s++*	Indole replaced with naphthalene alters shape and flexibility for improved interaction.	10.802	4.848
16	*Mor15i*, *map4_26*, *ETA_shape_p*, *R3s++*	Pyridone replacement with fused eight-membered ring adjusts shape and electronic characteristics.	11.628	4.840

**Table 3:** ADMET profiles of the compounds **15** and **16**.

Parameters	15	16
MW (g/mol)	465.18	437.18
Vol (Å^3^)	470.19	446.74
QED	0.603	0.585
Synth	3.8	3.204
Fsp3	0.346	0.32
logS (log mol/L)	−4.386	−4.965
logD	2.718	3.073
logP	2.417	3.229
DILI	0.835	0.926
Ames	0.438	0.433
FDAMDD	0.686	0.615
caco2 (cm/s)	−4.658	−4.906
PAMPA (cm/s)	0.09	0.016
hia	0	0
BBB	0.564	0.998

**Table 4:** Docking results of designed compounds for anticancer and anti-Alzheimer activity.

Ligand	Target	ΔG (kcal/mol)	Benchmark (ΔG)	Target Affinity Profile
**15**	AChE	−10.6	Donepezil (−11.8)	Strong Inhibitor
**15**	Tubulin	−9.7	Colchicine (−10.1)	Moderate Inhibitor
**16**	AChE	−9.7	Donepezil (−11.8)	Moderate Inhibitor
**16**	Tubulin	−10.0	Colchicine (−10.1)	Strong Inhibitor

## Data Availability

The data supporting the findings of this study, including all molecular structures, computational models, raw and processed trajectory data, and scripts required for the full replication of the integrated QSAR-MD-DCCM pipeline, are openly available in a public GitHub repository: https://github.com/tusharpawar49/QSAR-MD-DCCM_Dual-Target_Tubulin-AChE, accessed on 1 December 2025.

## Supplementary Materials

The following supporting information can be downloaded at: https://www.mdpi.com/article/10.3390/ph19020249/s1.
