# Supplementary material for: An Integrated QSAR-MD-DCCM Pipeline: A Predictive Computational Platform for the Rational Design and Dynamic Functional Validation of Dual-Target Directed Ligands
Source: Pharmaceuticals (Basel). 2026 Feb 1;19(2):249. doi: 10.3390/ph19020249 (PMC12943165; doi:10.3390/ph19020249)
Supplement: Supplementary file 1 [file pharmaceuticals-19-00249-s001.zip › Supplementary Information.pdf]

Supplementary Information

An Integrated QSAR-MD-DCCM Pipeline: A Predictive Computational Platform for the Rational Design and Dynamic Functional Validation of Dual-Target Directed Ligands

Shrikant S. Nilewar [1], Santosh Chobe [2], Prashik Dudhe [3], Perli Kranti Kumar [4], Sandesh Lodha [1], Akansha D. Raut [5], Dennys Fernández-Conde [6], Mohd Farhan [7], Ghazala Muteeb [8,*] and Tushar Janardan Pawar [6,9,*]

1 Department of Pharmaceutical Chemistry, Maliba Pharmacy College, Uka Tarsadia University,
Bardoli 394350, Gujrat, India; shrinilewar@gmail.com (S.S.N.); sandeshlodha@gmail.com (S.L.)

2 Department of Chemistry, M.G.V.'s Loknete Vyankatrao Hiray, Arts, Science and Commerce College,
Panchavati, Nashik 422003, Maharashtra, India; chobess222@gmail.com

3 School of Pharmaceutical Science, Sandip University, Nashik 422213, Maharashtra, India; dudhe.prashik@gmail.com

4 Department of Pharmaceutical Analysis, JKKN College of Pharmacy, Komarapalayam 638183, Tamil Nadu, India; drpkk1987@gmail.com

5 Department of Chemistry, School of science, Sandip University, Nashik 422213, Maharashtra, India;
rautakansha18@gmail.com

6 Dirección de Mecatrónica, Universidad Politécnica de Querétaro, Carretera Estatal 420 S/N, El Rosario 76240, Querétaro, Mexico; dennys.fernandez@upq.edu.mx

7 Department of Chemistry, College of Sciences, King Faisal University, Al-Ahsa 31982, Saudi Arabia;
mfarhan@kfu.edu.sa

8 Department of Nursing, College of Applied Medical Sciences, King Faisal University, Al-Ahsa 31982, Saudi Arabia

9 Escuela de Ingeniería Química, Universidad Anáhuac Querétaro, Circuito Universidades I, Fracción 2 S/N, Zibatá, El Marqués 76246, Querétaro, Mexico

* Correspondence: graza@kfu.edu.sa (G.M.); tushar.pawar@anahuac.mx (T.J.P.)

S1. Computational Tools, Software and workflow

Table S1: Comprehensive List of Computational Tools and Software

Sr. No	Tools	Application	Web link
1	ChEMBL	Literature review and structure retrieval	https://www.ebi.ac.uk/chembl/
2	Bindingdb		https://www.bindingdb.org/
3	QSARINS	QSAR modelling	https://dunant.dista.uninsubria.it/qsar/?page_id=460
4	Marvin sketch	Ligand sketching and optimization	https://chemaxon.com/marvin
5	RCSB:PDB	Protein database	https://www.rcsb.org/
6	CB-Dock2	Protein-ligand docking tool	http://cadd.labshare.cn/cb-dock2/index.php
7	AutoDock Vina	Docking module used in CB-Dock2	https://vina.scripps.edu/
8	DockRMSD	Docking validation	https://zhanggroup.org/DockRMSD/
9	Discovery studio visualizer	2D interaction analysis	https://discover.3ds.com/discovery-studiovisualizer-download
10	Swisspdb Viewer	Preparation of Protein	https://spdbv.unil.ch/contactSurf_tut.html
11	Protein Plus	3D binding pocket visualizer	https://proteins.plus/
12	ADMETlab 3.0	ADMET profiling of the candidates	https://admetlab3.scbdd.com/
13	ProTox	Toxicity profiling	https://tox.charite.de/protox3/
14	Desmond/Schrodinger Suit	Molecular dynamic simulation studies	https://www.schrodinger.com/products/maestro
15	Ramachandran Plot	Post_MD stability analysis	https://molprobity.biochem.duke.edu/index.php
16	MD TASK	Dynamic cross correlation matrix	https://bioinformaticshome.com/db/tool/MD-TASK

S2. QSAR Model Validation and Data

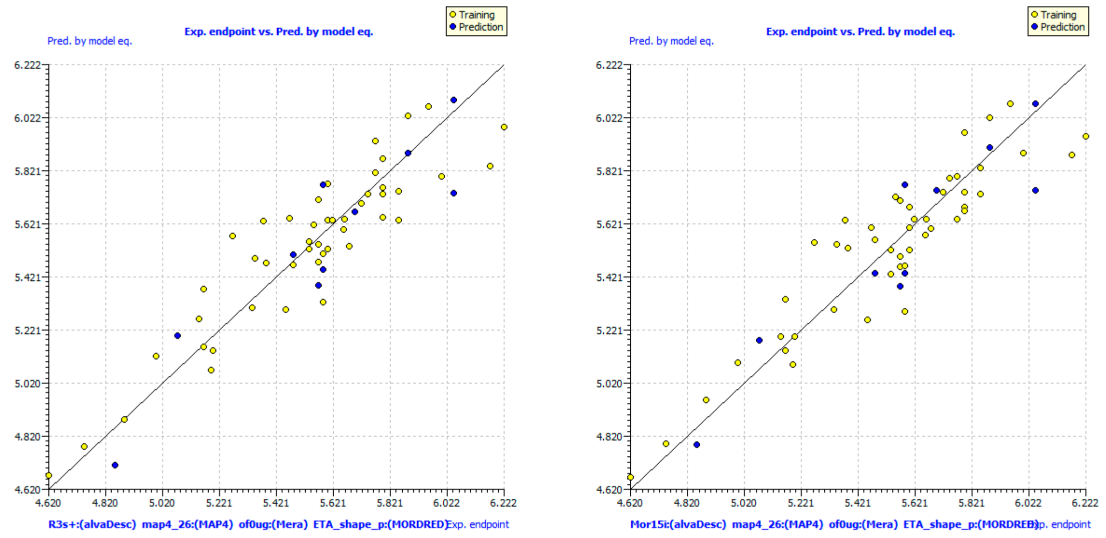

Model 1 **Model 2**

Figure S1a: Experimental Endpoint vs. Predicted by model.

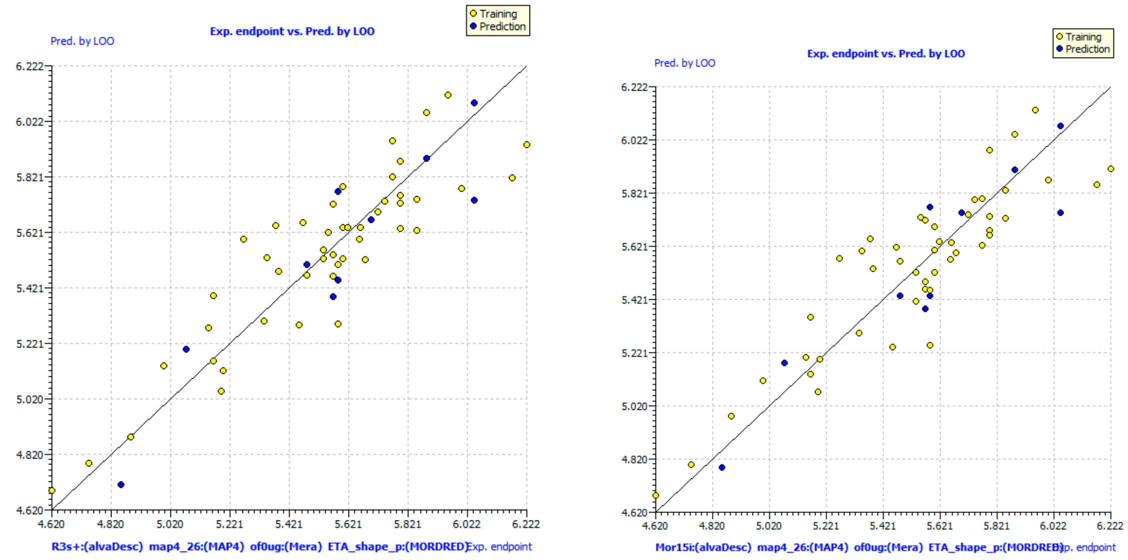

Model 1 **Model 2**

Figure S1b: Experimental Endpoint vs. Predicted by LOO.

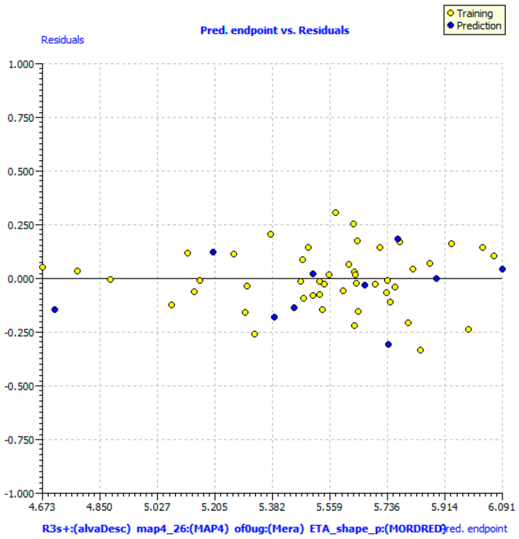

Model 1

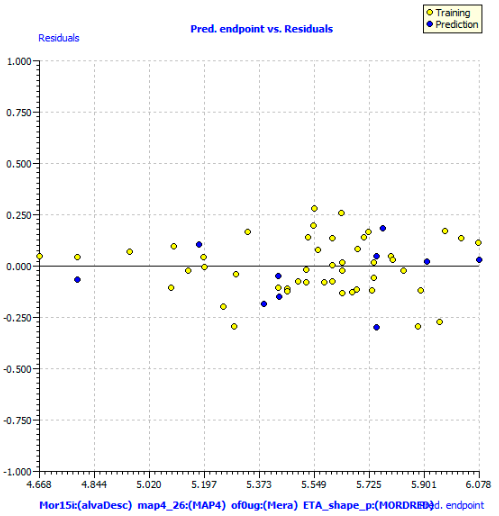

Model 2

Figure S1c: Residuals predicted by model equation.

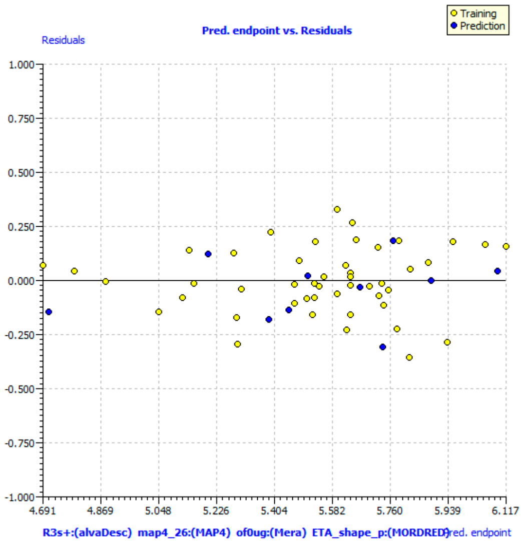

Model 1

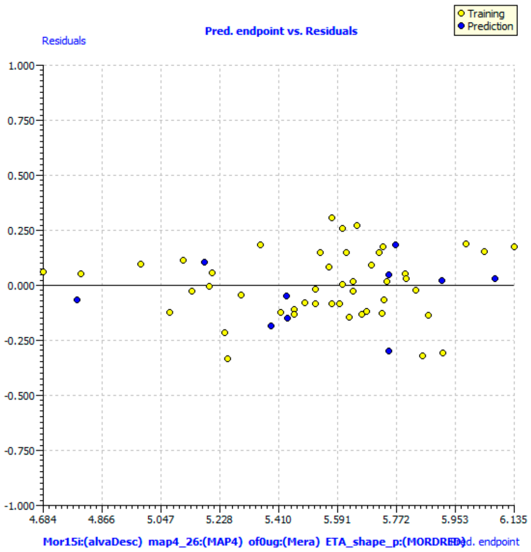

Model 2

Figure S1d: Residuals predicted by LOO.

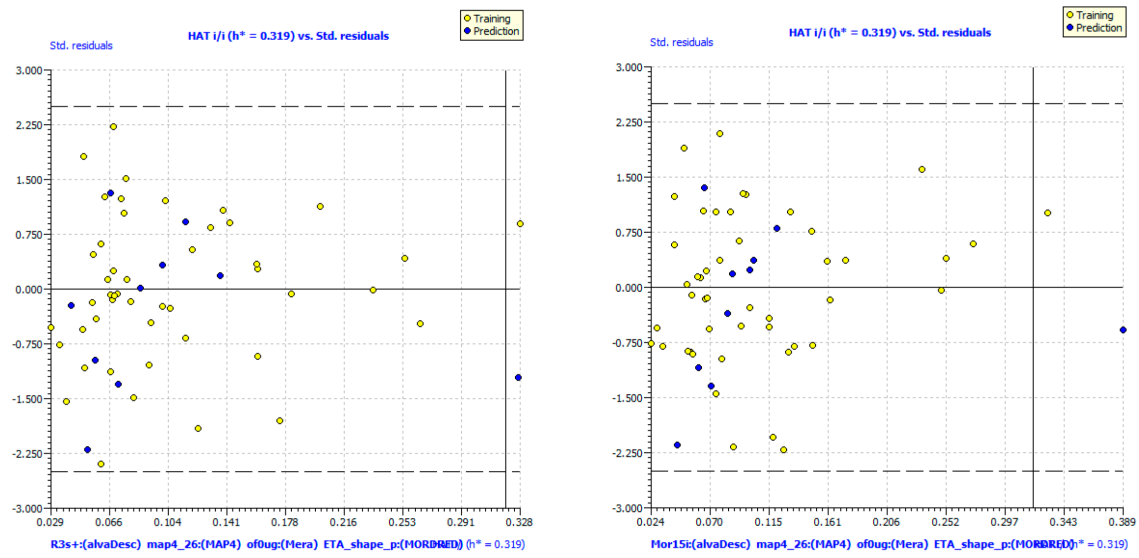

Model 1 **Model 2**

Figure S1e: Williams plot- predictions by model equation.

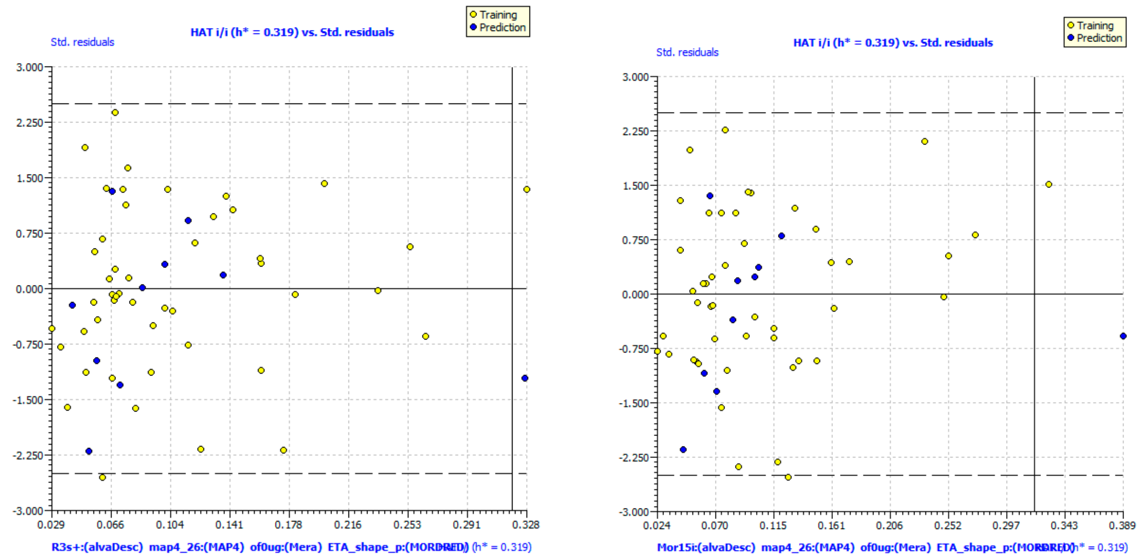

Model 1 **Model 2**

Figure S1f: Williams plot- predictions by LOO.

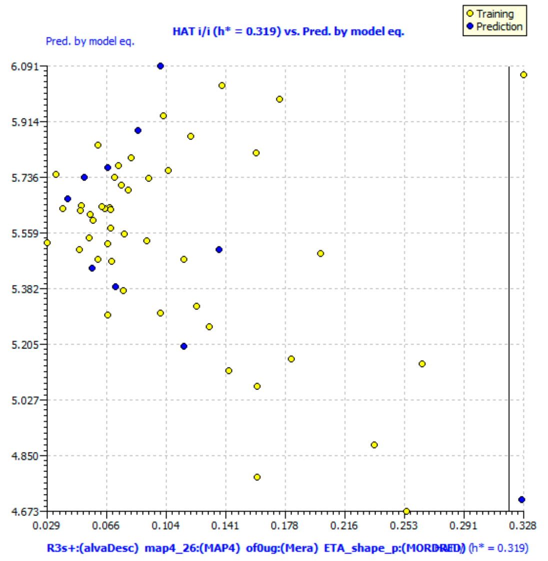

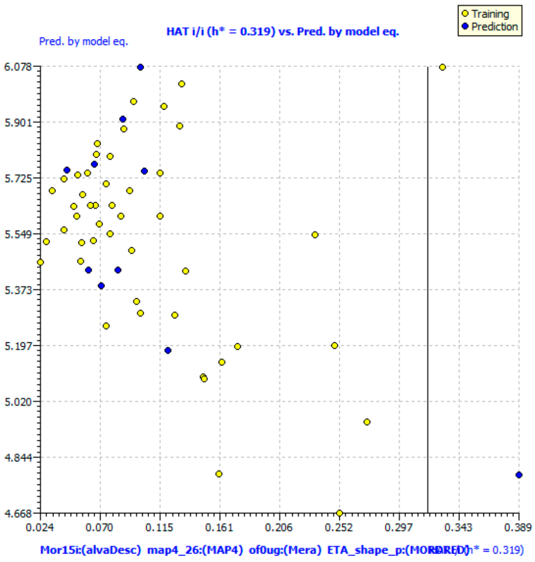

Model 1 **Model 2**

Figure S1g: Applicability Domain.

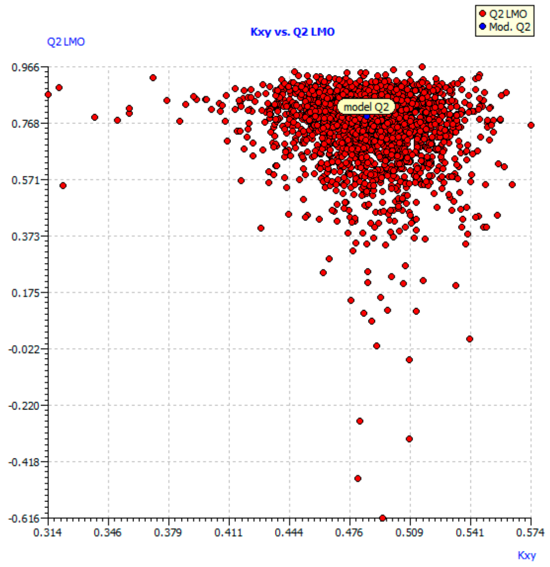

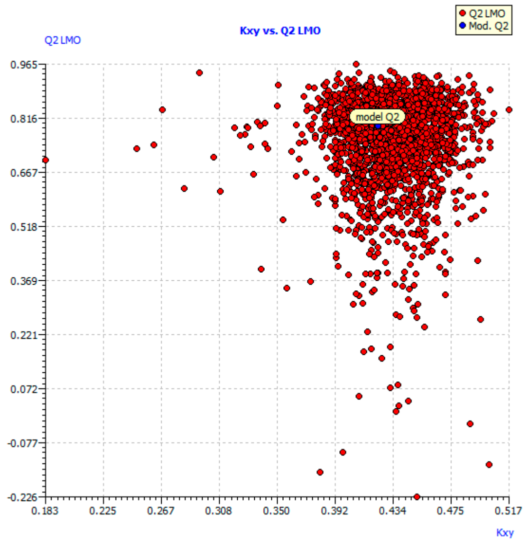

Model 1 **Model 2**

Figure S1h: LMO Plot.

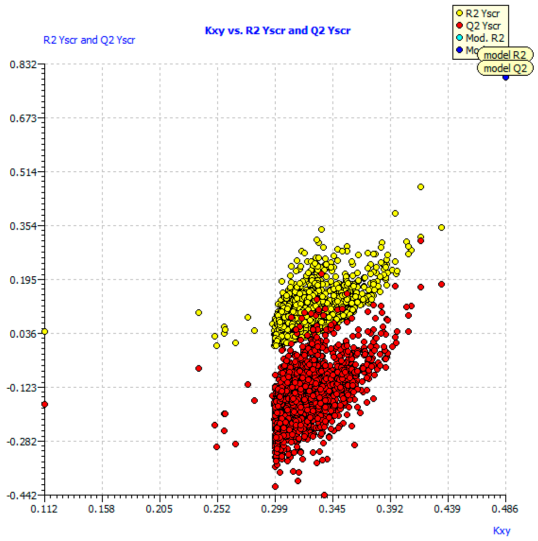

Model 1

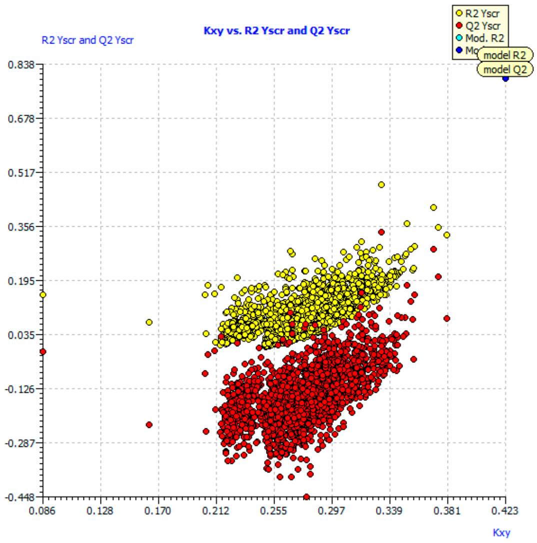

Model 2

Figure S1i: Y-scramble Plot.

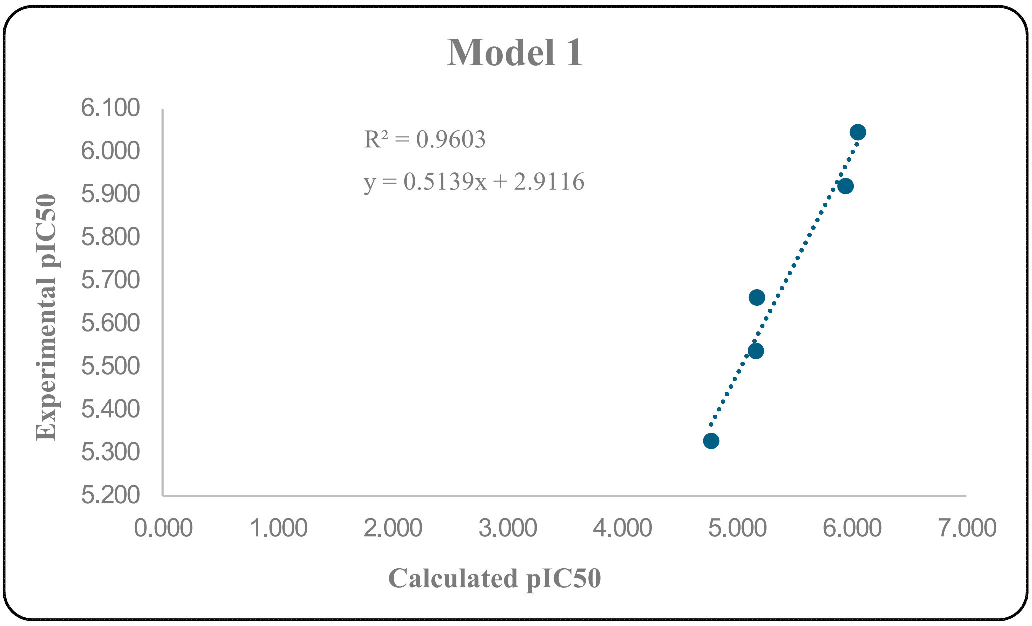

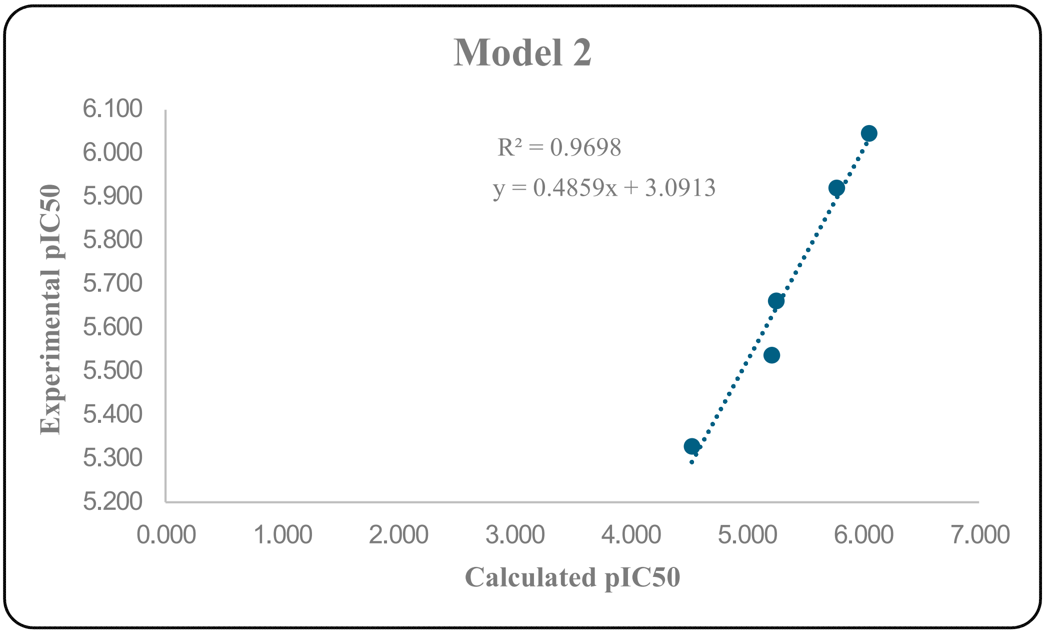

Figure S2: External validation graphs of generated models

QSAR Descriptor Definitions

Table S2: Definitions and Mechanistic Interpretation of Key Molecular Descriptors.

Descriptor	Full Definition and Origin	Mechanistic Interpretation in Models
map4_26	Topological Complexity/Molecular Architecture. Derived from the MAP4 (Molecular ACCessible Surface Area Property) fingerprint. It represents atom-pair interactions and branching patterns within the molecular topology.	Its positive contribution suggests that a precise degree of molecular complexity and a specific branching structure are required for optimal fit and recognition within the constrained colchicine binding pocket.
of0ug	Molecular Polarizability. An electronic descriptor (MERA) that quantifies the ability of a molecule to redistribute its electron density when subjected to an electric field.	It is the most dominant determinant (highest coefficient) in both models. A high positive value indicates that high electronic polarizability is essential for strong activity, facilitating favorable non-covalent, van der Waals, and induced dipole interactions with the dynamic tubulin structure.
ETA_shape_p	3D Conformational Flexibility/Shape Adaptability. An electronic-topological descriptor (MORDRED) that evaluates the responsiveness of a molecule's three-dimensional shape to conformational changes.	Its strong positive coefficient confirms that a certain level of molecular flexibility and shape adaptability is advantageous, enabling the ligand to optimally adjust its conformation to fit the dynamic binding pockets of tubulin during polymerization inhibition.
R3s++	Steric Accessibility. A 3D descriptor (alvaDesc) that assesses the degree of steric hindrance and the spatial accessibility around specific atoms or regions of the molecule.	Unique to Model 1, its positive contribution confirms that reduced steric hindrance and a shape optimized for easy access are critical prerequisites for effective binding within the spatially confined colchicine pocket.
Mor15i	Electronic Density/Polarizability Influence. A 3D-MoRSE (Molecular Representation of Structures based on Electronic Distribution) descriptor. It focuses on the influence of electronic density and atomic masses.	Unique to Model 2, its inclusion reinforces the importance of electronic complementarity and specific mass distribution in fine-tuning the inhibitory potential, complementing the broad of0ug metric.

Full Dataset:

Table S3: 57 original molecules used for model training and validation. SMILES, the experimental IC_{50}, and the calculated pIC_{50} value. Detailed data available at: https://github.com/tusharpawar49/QSAR-MD-DCCM_Dual-Target_Tubulin-AChE/

Sr. No.	Compounds	Status	Experimental IC_{50} (µM)	Calculated pIC_{50}
**S1**	COC1=CC(NC(=O)C(=C\C2=CNC3=C2C=CC=C3)\C#N)=CC(OC)=C1OC	Training	18.0	4.744727
**S2**	COC1=CC=C2C(NC=C2C(=O)C2=CC(OC)=C(OC)C(OC)=C2)=C1	Training	2.2	5.661544
**S3**	COC1=CC=C2C(NC(C)=C2C(=O)C2=CC(OC)=C(OC)C(OC)=C2)=C1	Prediction	0.9	6.045757
**S4**	COC1=CC=C2N(C=CC2=C1N)C(=O)C1=CC(OC)=C(OC)C(OC)=C1	Prediction	0.9	6.045757
**S5**	COC1=CC=C2N(C(=O)C3=CC(OC)=C(OC)C(OC)=C3)C(C)=CC2=C1O	Training	0.6	6.221849
**S6**	COC1=CC(=CC(OC)=C1OC)C(=O)C1=CC2=C(C=C1)N(C)C=C2	Training	1.1	5.958607
**S7**	COC1=CC(=CC(OC)=C1OC)C(=O)C1=C(N)C2=C(C=CC(C)=C2)N1C	Training	10.0	5.00000
**S8**	COC1=CC(=CC(OC)=C1OC)C(=O)C1=C(N)C2=C(C=C(C)C=C2)N1C	Training	6.4	5.19382
**S9**	COC1=CC(=CC(OC)=C1OC)C(=O)C1=C(N)C2=C(N1C)C(C)=CC=C2	Prediction	8.4	5.075721
**S10**	COC1=CC2=C(C=C1)C(N)=C(N2C)C(=O)C1=CC(OC)=C(OC)C(OC)=C1	Training	4.6	5.337242
**S11**	COC1=CC(=CC(OC)=C1OC)C(=O)C1=C(N)C2=C(C=C(Cl)C=C2)N1C	Training	7.1	5.148742
**S12**	COC1=CC(=CC(OC)=C1OC)C(=NO)C1=CC2=C(C=C1)N(C)C=C2	Training	1.8	5.744727
**S13**	COC(=O)C1=C(SC2=CC(OC)=C(OC)C(OC)=C2)C2=C(N1)C=CC=C2	Training	2.9	5.537602
**S14**	COC(=O)C1=C(SC2=CC(OC)=C(OC)C(OC)=C2)C2=C(N1)C=CC(Cl)=C2	Training	2.5	5.60206
**S15**	COC(=O)C1=C(SC2=CC(OC)=C(OC)C(OC)=C2)C2=C(N1)C=CC(OC)=C2	Prediction	2.0	5.69897
**S16**	CCOC(=O)C1=C(SC2=CC(OC)=C(OC)C(OC)=C2)C2=C(N1)C=CC=C2	Training	2.9	5.537602
**S17**	CCOC(=O)C1=C(SC2=CC(OC)=C(OC)C(OC)=C2)C2=C(N1)C=CC(Cl)=C2	Training	2.2	5.657577
**S18**	CCOC(=O)C1=C(SC2=CC(OC)=C(OC)C(OC)=C2)C2=C(N1)C=CC(OC)=C2	Training	2.4	5.619789
**S19**	COC(=O)C1=C(SC2=CC(OC)=C(OC)C(OC)=C2)C2=C(N1)C=CC(=C2)[N+]([O-])=O	Training	4.5	5.346787
**S20**	COC1=CC(SC2=CNC3=C2C=CC=C3)=CC(OC)=C1OC	Training	2.6	5.585027
**S21**	COC1=CC(SC2=C(C)NC3=C2C=CC=C3)=CC(OC)=C1OC	Training	6.8	5.167491

**S22**	COC1=CC(SC2=CNC3=C2C=C(Cl)C=C3)=CC(OC)=C1OC	Prediction	2.6	5.585027
**S23**	COC1=CC(SC2=C(C)NC3=C2C=C(Cl)C=C3)=CC(OC)=C1OC	Training	2.7	5.568636
**S24**	COC1=CC(SC2=CNC3=C2C=C(Br)C=C3)=CC(OC)=C1OC	Training	1.6	5.79588
**S25**	COC1=CC(SC2=CNC3=C2C=C(I)C=C3)=CC(OC)=C1OC	Training	2.7	5.568636
**S26**	COC1=CC(SC2=CNC3=C2C=C(F)C=C3)=CC(OC)=C1OC	Prediction	3.3	5.481486
**S27**	COC1=CC(SC2=CNC3=C2C=C(C)C=C3)=CC(OC)=C1OC	Prediction	2.7	5.568636
**S28**	COC1=CC2=C(NC=C2SC2=CC(OC)=C(OC)C(OC)=C2)C=C1	Training	4.1	5.387216
**S29**	COC1=CC2=C(NC(C)=C2SC2=CC(OC)=C(OC)C(OC)=C2)C=C1	Training	3.3	5.481486
**S30**	CCOC1=CC2=C(NC=C2SC2=CC(OC)=C(OC)C(OC)=C2)C=C1	Training	2.1	5.677781
**S31**	COC1=CC(SC2=CNC3=C2C=C(O)C=C3)=CC(OC)=C1OC	Training	6.3	5.200659
**S32**	COC1=CC(SC2=CNC3=C2C=C(OCCO)C=C3)=CC(OC)=C1OC	Training	6.8	5.167491
**S33**	COC1=CC(=CC(OC)=C1OC)C(=O)C1=CNC2=C1C=CC=C2	Training	3.5	5.455932
**S34**	COC(=O)C1=C(C(=O)C2=CC(OC)=C(OC)C(OC)=C2)C2=C(N1)C=CC=C2	Training	2.7	5.568636
**S35**	CCOC(=O)C1=C(C(=O)C2=CC(OC)=C(OC)C(OC)=C2)C2=C(N1)C=CC=C2	Training	2.6	5.585027
**S36**	COC1=CC(=CC(OC)=C1OC)C(=O)C1=CNC2=C1C=C(Cl)C=C2	Training	2.5	5.60206
**S37**	COC(=O)C1=C(C(=O)C2=CC(OC)=C(OC)C(OC)=C2)C2=C(N1)C=CC(Cl)=C2	Training	1.6	5.79588
**S38**	CCOC(=O)C1=C(C(=O)C2=CC(OC)=C(OC)C(OC)=C2)C2=C(N1)C=CC(Cl)=C2	Training	1.4	5.853872
**S39**	COC1=CC(CC2=CNC3=C2C=C(Cl)C=C3)=CC(OC)=C1OC	Training	24.0	4.619789
**S40**	COC(=O)C1=C(CC2=CC(OC)=C(OC)C(OC)=C2)C2=C(N1)C=CC=C2	Training	4.2	5.376751
**S41**	CCOC(=O)C1=C(CC2=CC(OC)=C(OC)C(OC)=C2)C2=C(N1)C=CC=C2	Training	5.4	5.267606
**S42**	COC(=O)C1=C(CC2=CC(OC)=C(OC)C(OC)=C2)C2=C(N1)C=CC(Cl)=C2	Training	1.7	5.769551
**S43**	CCOC(=O)C1=C(CC2=CC(OC)=C(OC)C(OC)=C2)C2=C(N1)C=CC(Cl)=C2	Training	2.5	5.60206
**S44**	COC1=CC(=CC(OC)=C1OC)C(=O)C1=CNC2=C1C=C(Br)C=C2	Training	1.9	5.721246
**S45**	COC1=CC(CC2=CNC3=C2C=C(Br)C=C3)=CC(OC)=C1OC	Training	13.0	4.886057
**S46**	COC(=O)C1=C(SC2=CC(OC)=C(OC)C(OC)=C2)C2=C(N1)C=CC(Br)=C2	Training	1.0	6.004365
**S47**	COC(=O)C1=C(C(=O)C2=CC(OC)=C(OC)C(OC)=C2)C2=C(N1)C=CC(Br)=C2	Training	1.3	5.886057
**S48**	COC(=O)C1=C(CC2=CC(OC)=C(OC)C(OC)=C2)C2=C(N1)C=CC(Br)=C2	Prediction	1.3	5.886057

**S49**	CCOC(=O)C1=C(SC2=CC(OC)=C(OC)C(OC)=C2)C2=C(N1)C=CC(Br)=C2	Training	1.6	5.79588
**S50**	CCOC(=O)C1=C(C(=O)C2=CC(OC)=C(OC)C(OC)=C2)C2=C(N1)C=CC(Br)=C2	Training	1.6	5.79588
**S51**	CCOC(=O)C1=C(CC2=CC(OC)=C(OC)C(OC)=C2)C2=C(N1)C=CC(Br)=C2	Training	1.7	5.769551
**S52**	COC1=CC2=C(NC=C2C(=O)C2=CC(OC)=C(OC)C(OC)=C2)C=C1	Training	3.4	5.468521
**S53**	COC1=CC2=C(NC=C2CC2=CC(OC)=C(OC)C(OC)=C2)C=C1	Prediction	14.0	4.853872
**S54**	COC(=O)C1=C(C(=O)C2=CC(OC)=C(OC)C(OC)=C2)C2=C(N1)C=CC(OC)=C2	Training	0.7	6.173925
**S55**	COC(=O)C1=C(CC2=CC(OC)=C(OC)C(OC)=C2)C2=C(N1)C=CC(OC)=C2	Training	1.4	5.853872
**S56**	CCOC(=O)C1=C(C(=O)C2=CC(OC)=C(OC)C(OC)=C2)C2=C(N1)C=CC(OC)=C2	Prediction	2.6	5.585027
**S57**	CCOC(=O)C1=C(CC2=CC(OC)=C(OC)C(OC)=C2)C2=C(N1)C=CC(OC)=C2	Training	2.8	5.552842

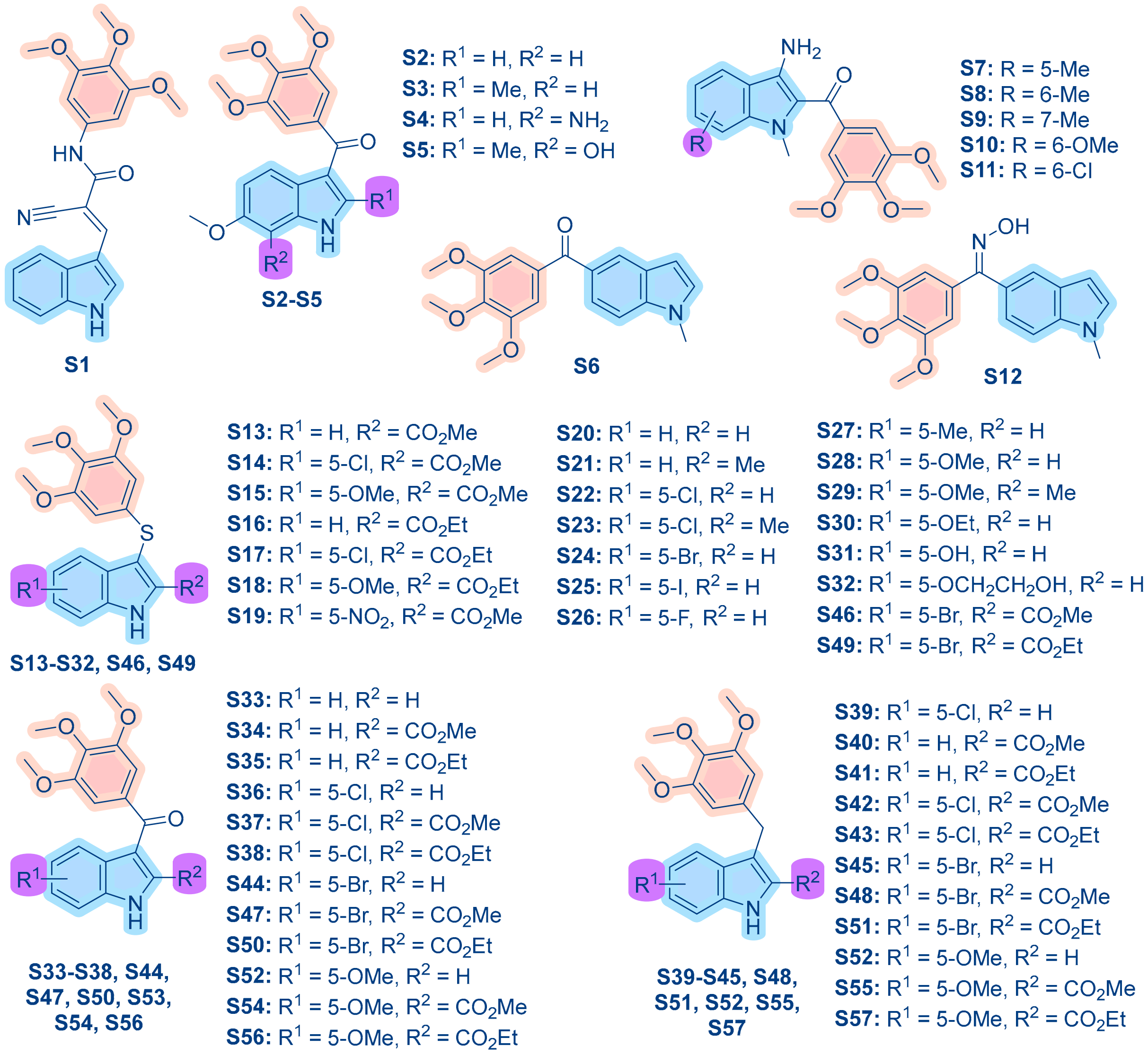

Figure S3: Chemical Structures of 57 Compounds used for QSAR.

S3. Molecular Design and ADMET Profiles

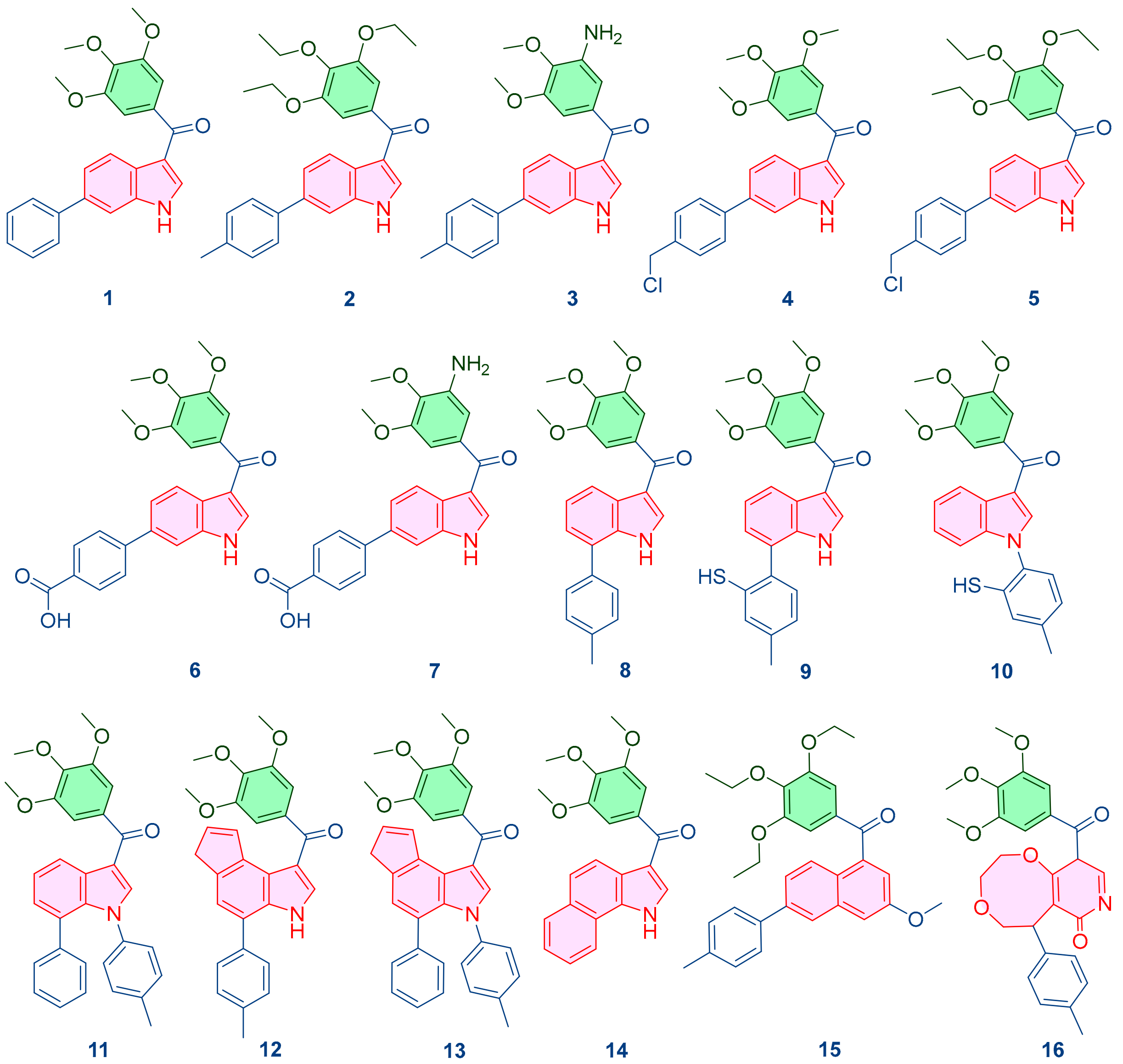

Figure S4: Full Chemical Structures of Designed Compounds

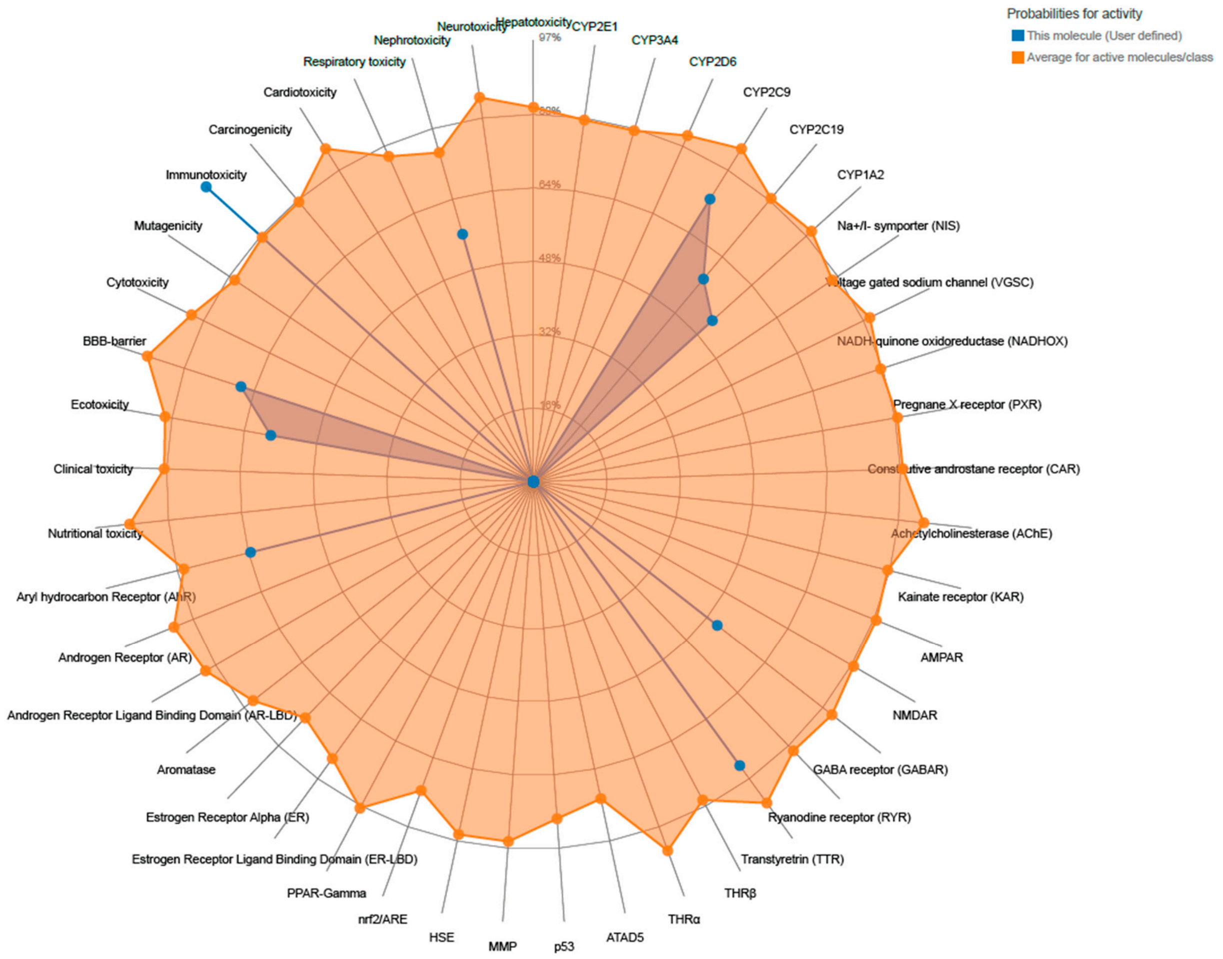

Figure S5a: Toxicity profile of Compound **15.**

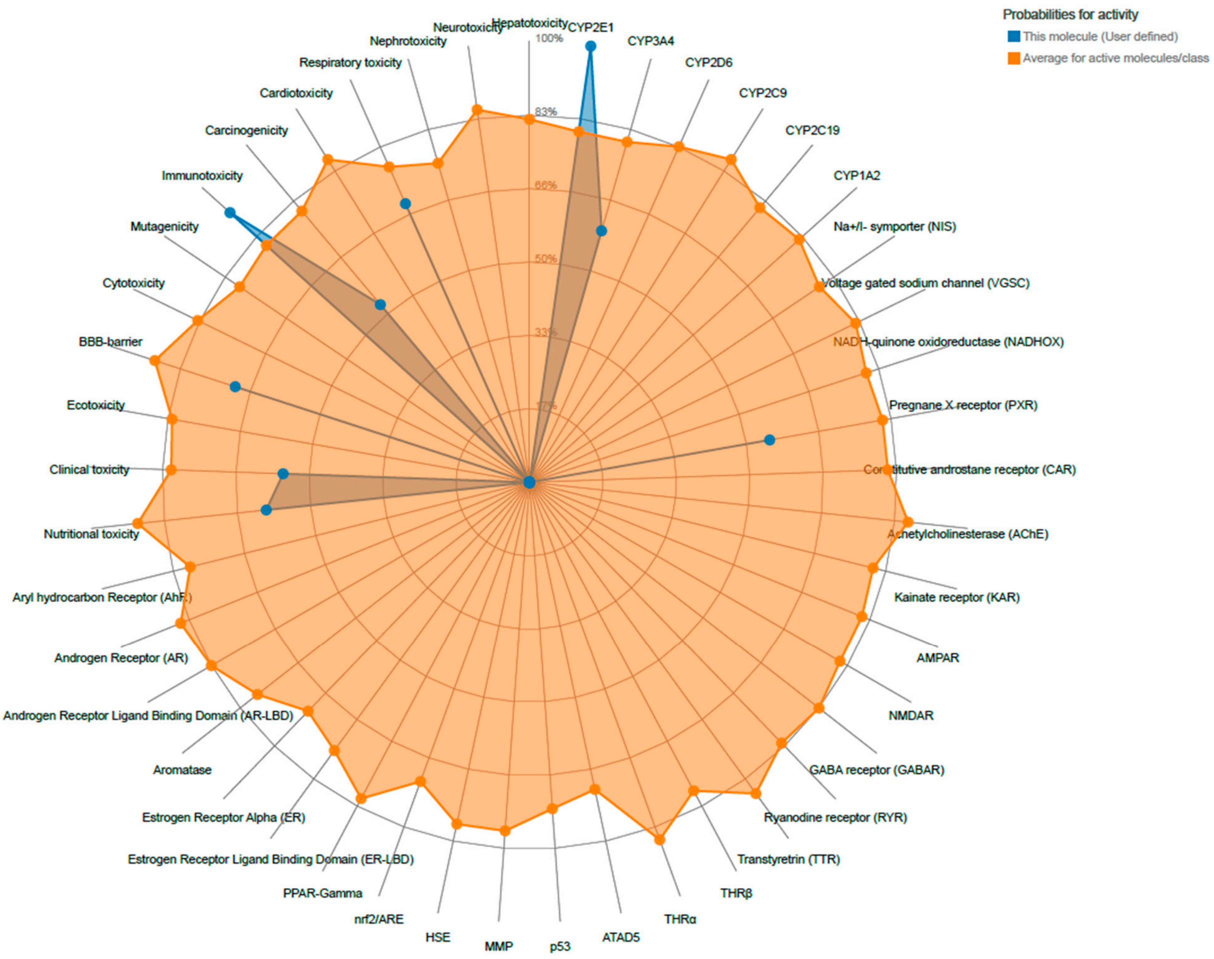

Figure S5b: Toxicity profile of Compound **16.**

Table S4: ADMET profiles of the designed compounds.

Parameters	14	3	5	1	2	6	7	4	8	9	10	11	12	13	**15**	**16**
MW	484.220	443.210	386.160	435.120	477.170	431.140	416.140	401.160	433.130	477.190	439.180	515.210	361.130	387.150	**465.180**	**437.180**
Vol	522.820	475.775	408.797	439.098	490.986	438.831	423.741	423.887	442.396	511.197	464.582	551.892	374.635	406.591	**470.190**	**446.740**
QED	0.222	0.297	0.375	0.293	0.197	0.408	0.318	0.440	0.303	0.240	0.376	0.212	0.529	0.468	**0.603**	**0.585**
Synth	2.343	2.302	2.279	2.277	2.434	2.202	2.339	2.202	2.552	2.341	2.765	2.819	2.194	2.079	**3.800**	**3.204**
Fsp3	0.258	0.250	0.125	0.160	0.250	0.120	0.083	0.160	0.160	0.129	0.179	0.147	0.136	0.125	**0.346**	**0.320**
logS	-8.469	-7.311	-6.296	-6.537	-7.145	-5.230	-5.158	-6.338	-6.070	-7.309	-6.837	-7.719	-5.832	-5.759	**-4.386**	**-4.965**
logD	5.152	4.436	3.206	3.530	4.179	2.645	2.459	3.749	3.642	4.552	3.738	4.548	3.494	3.569	**2.718**	**3.073**
logP	6.644	5.656	4.173	4.193	5.185	3.806	3.691	4.527	4.301	5.407	4.710	5.765	3.948	4.167	**2.417**	**3.229**
DILI	0.994	0.977	0.958	0.892	0.951	0.992	0.993	0.941	0.999	0.989	0.962	0.996	0.952	0.950	**0.835**	**0.926**
Ames	0.621	0.476	0.788	0.826	0.717	0.629	0.787	0.611	0.759	0.713	0.742	0.874	0.746	0.638	**0.438**	**0.433**
FDAMDD	0.866	0.837	0.859	0.837	0.864	0.685	0.761	0.792	0.271	0.818	0.852	0.913	0.795	0.793	**0.686**	**0.615**
caco2	-4.647	-4.736	-5.016	-4.962	-4.714	-5.034	-5.166	-4.990	-5.140	-4.899	-4.853	-4.804	-4.909	-4.958	**-4.658**	**-4.906**
PAMPA	0.099	0.191	0.237	0.013	0.033	0.800	0.932	0.010	0.002	0.006	0.012	0.010	0.008	0.040	**0.090**	**0.016**
hia	0.000	0.000	0.000	0.001	0.000	0.002	0.006	0.000	0.000	0.000	0.000	0.000	0.001	0.000	**0.000**	**0.000**
BBB	0.110	0.283	0.358	0.722	0.283	0.026	0.006	0.923	0.257	0.973	0.941	0.981	0.913	0.590	**0.564**	**0.998**

S4. Molecular Docking Details

Table S5: Docking validity evaluation

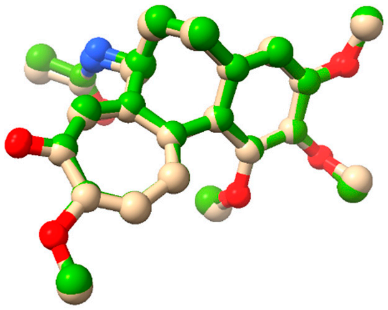

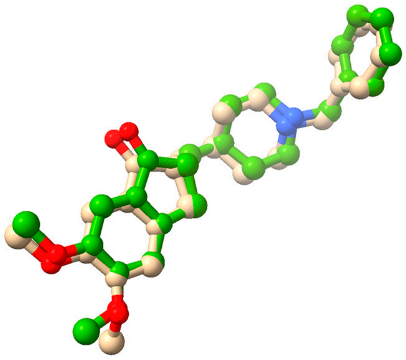

Ligand:Protein	Colchicine:Tubulin	Donepezil:AChE
RMSD	0.172	0.532
Legend	**Green:** Ligand obtained from Crystal<br>**Brown:** Docked ligand	**Green:** Ligand obtained from Crystal<br>**Brown:** Docked ligand

Table S6: Docking results of designed compounds for anticancer and anti-Alzheimer activity.

**Ligands**	**Binding Affinity (ΔG) (kcal/mol)**	
	**for Anticancer Activity**	**For Anti Alzheimer's Activity**
**15**	-9.7	-10.6
**16**	-10.0	-9.7
Colchicine	-10.1	-
Donepezil	-	-11.8

Compound 15-AChE

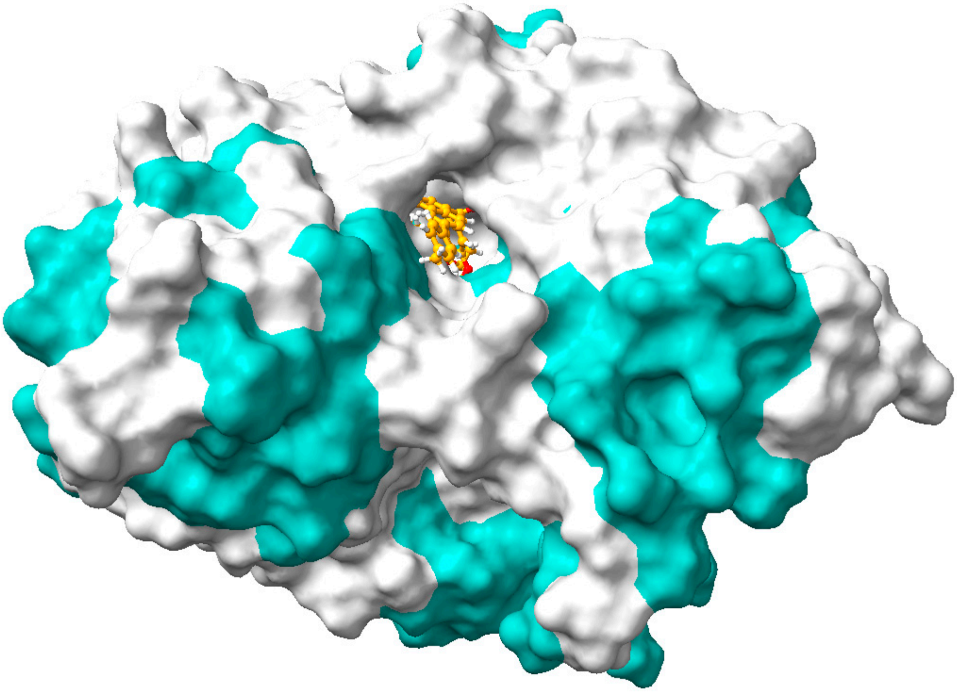

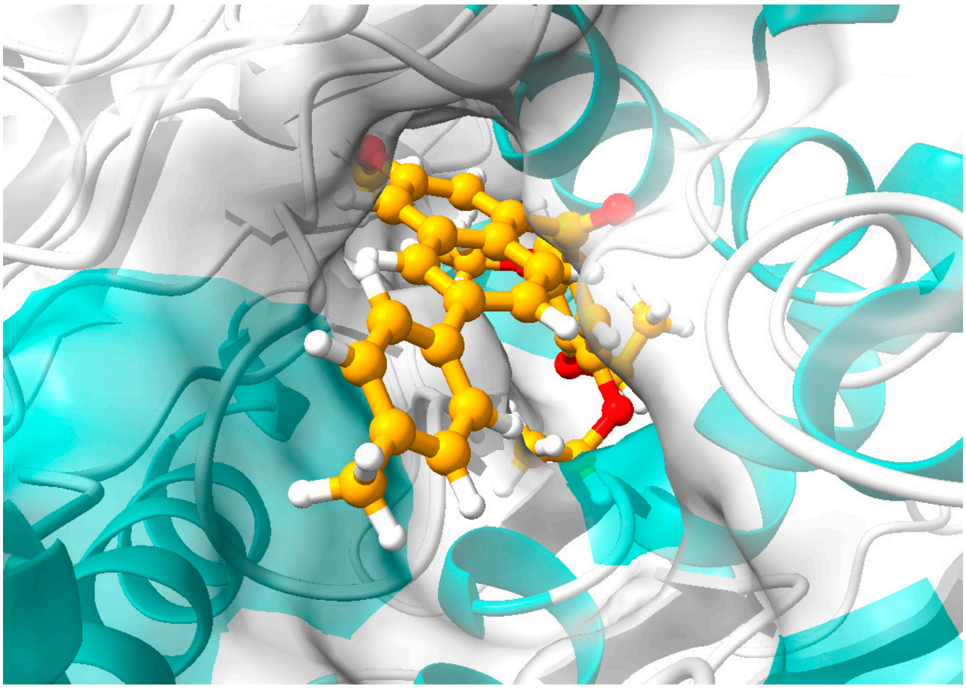

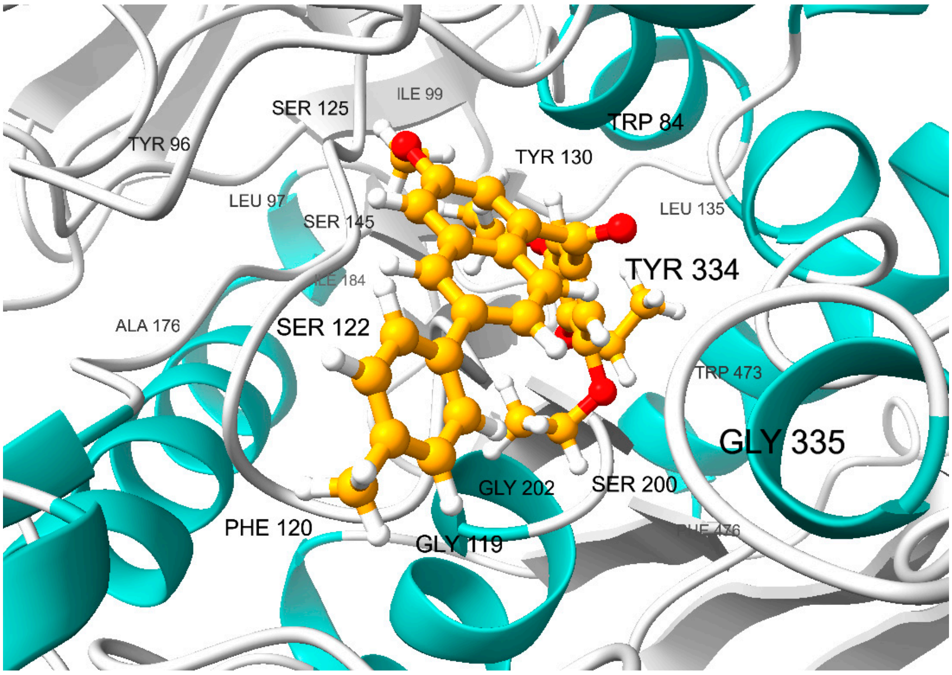
ILE 99
SER 125
TRP 84
TYR 96
TYR 130
LEU 97
SER 145
LEU 135
TYR 334
ALA 176
SER 122
TRP 473
GLY 335
GLY 202
SER 200
PHE 120
GLY 119

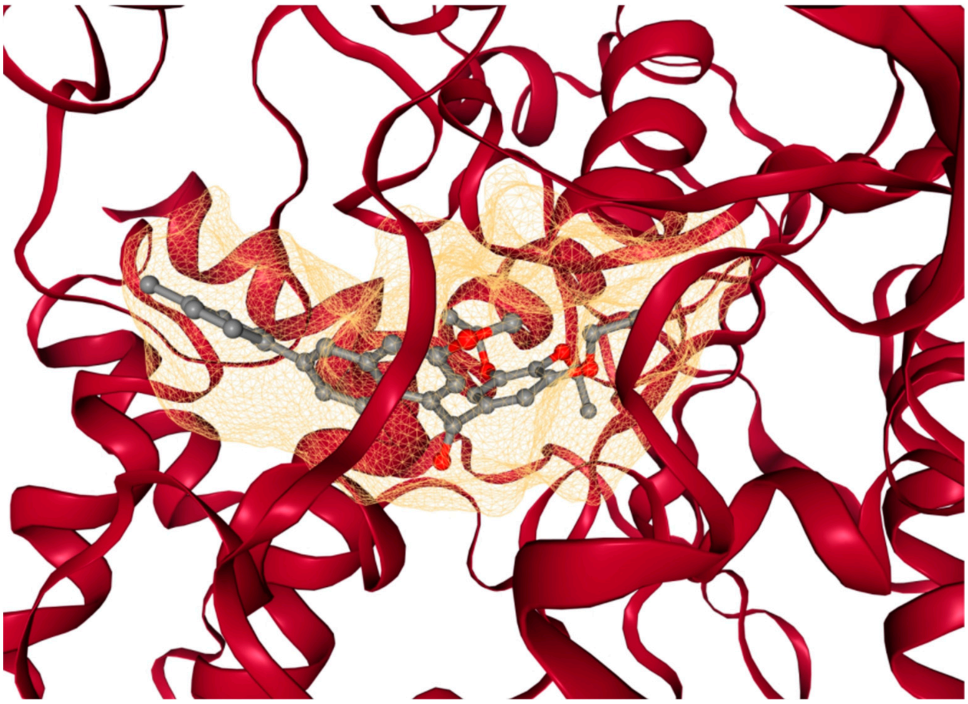

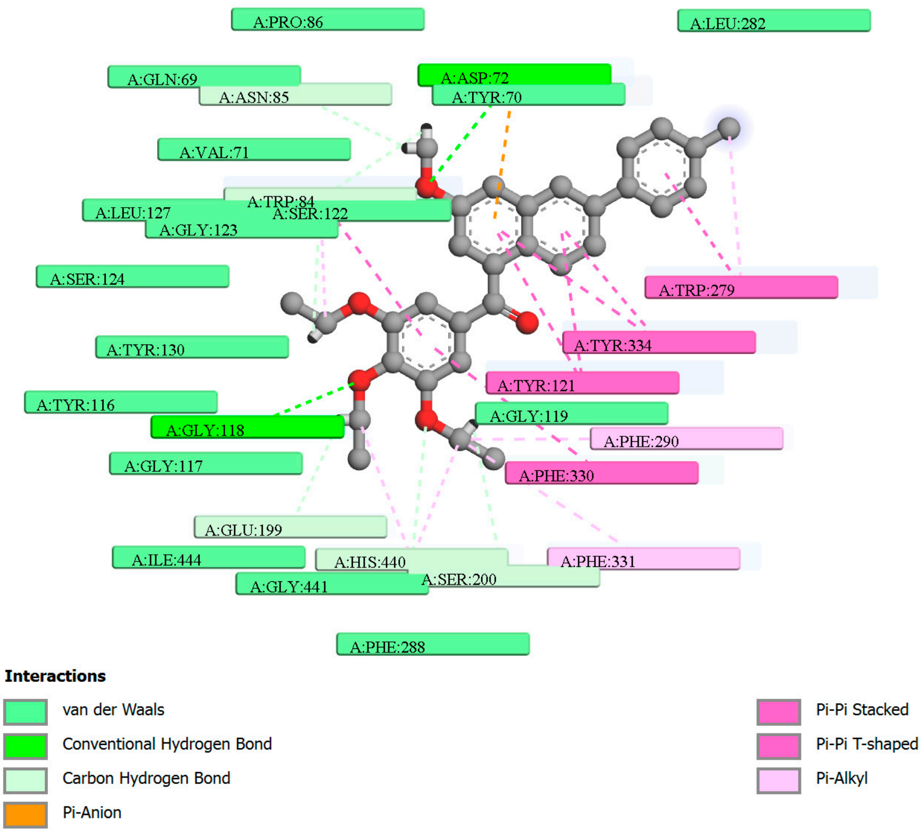
A:PRO:86
A:LEU:282
A:GLN:69
A:ASN:85
A:ASP:72
A:TYR:70
A:VAL:71
A:TRP:84
A:LEU:127
A:SER:122
A:GLY:123
A:SER:124
A:TRP:279
A:TYR:334
A:TYR:130
A:TYR:121
A:TYR:116
A:GLY:119
A:GLY:118
A:PHE:290
A:GLY:117
A:PHE:330
A:GLU:199
A:ILE:444
A:HIS:440
A:PHE:331
A:SER:200
A:GLY:441
A:PHE:288
Interactions
van der Waals
Conventional Hydrogen Bond
Carbon Hydrogen Bond
Pi-Anion
Pi-Pi Stacked
Pi-Pi T-shaped
Pi-Alkyl

Compound 16 -AChE

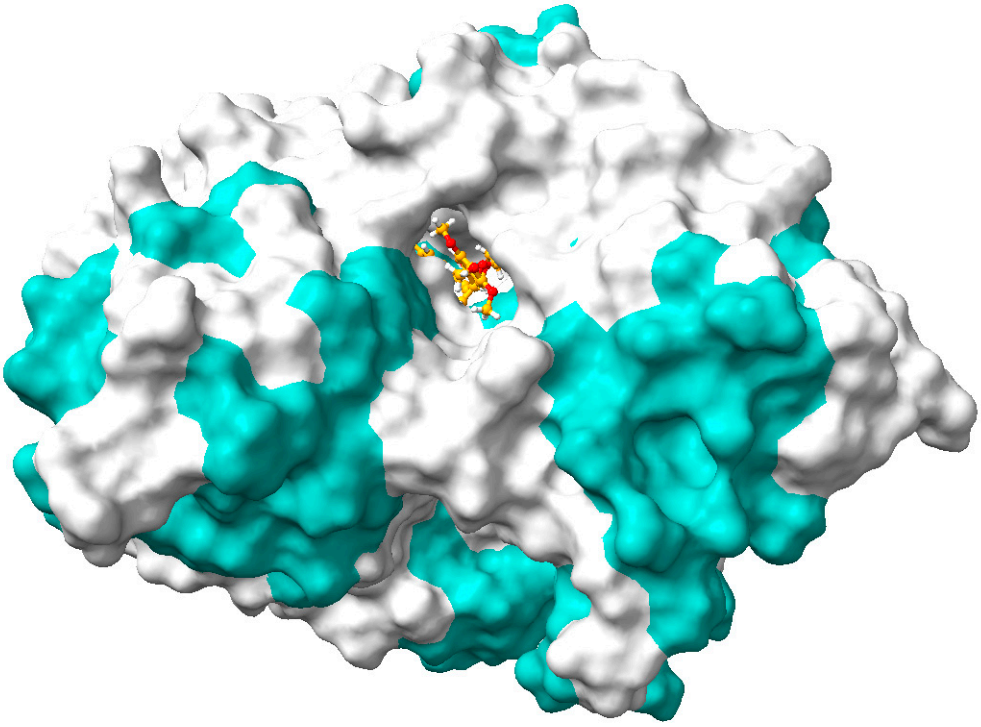

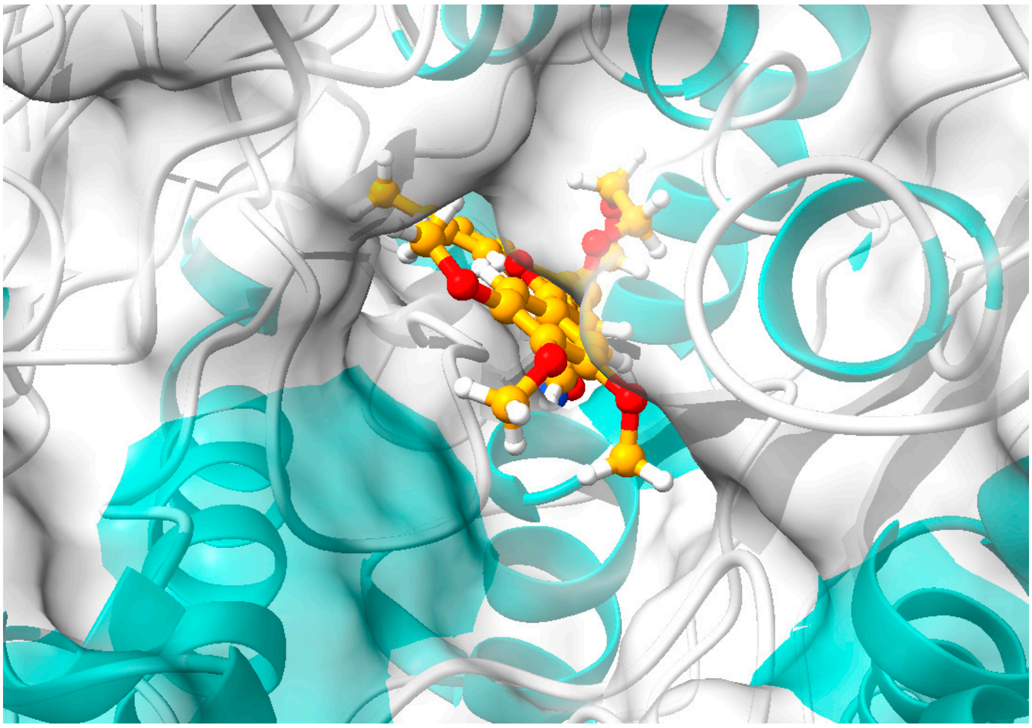

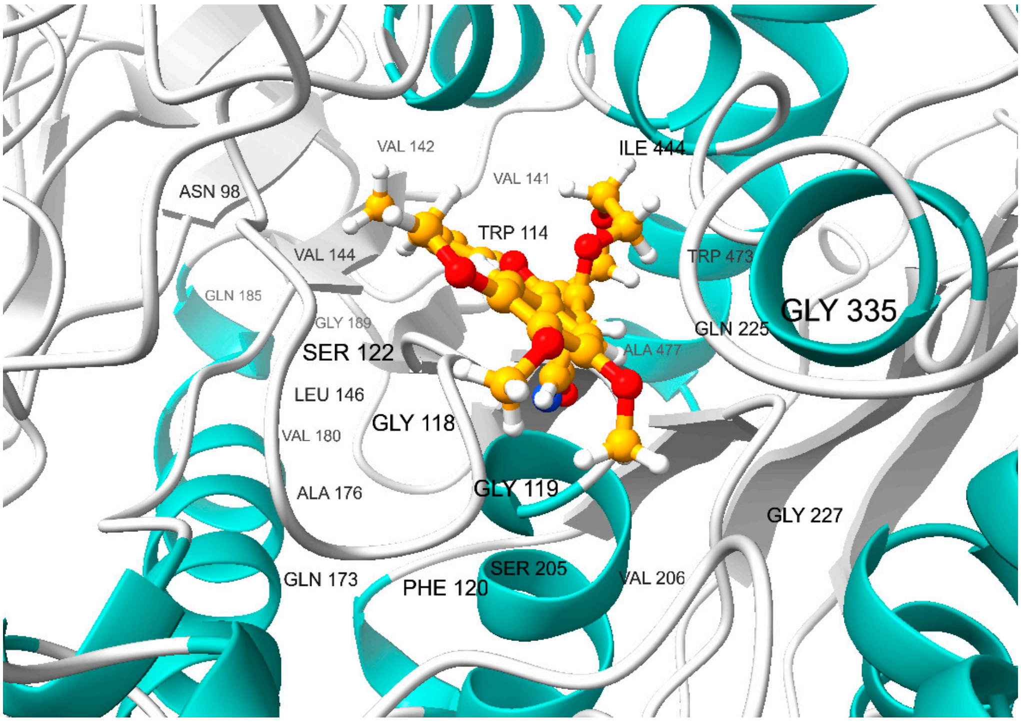
VAL 142
ILE 444
ASN 98
VAL 141
TRP 114
VAL 144
TRP 473
GLN 185
GLY 189
GLY 335
GLN 225
SER 122
ALA 477
LEU 146
GLY 118
VAL 180
ALA 176
GLY 119
GLY 227
GLN 173
PHE 120
SER 205
VAL 206

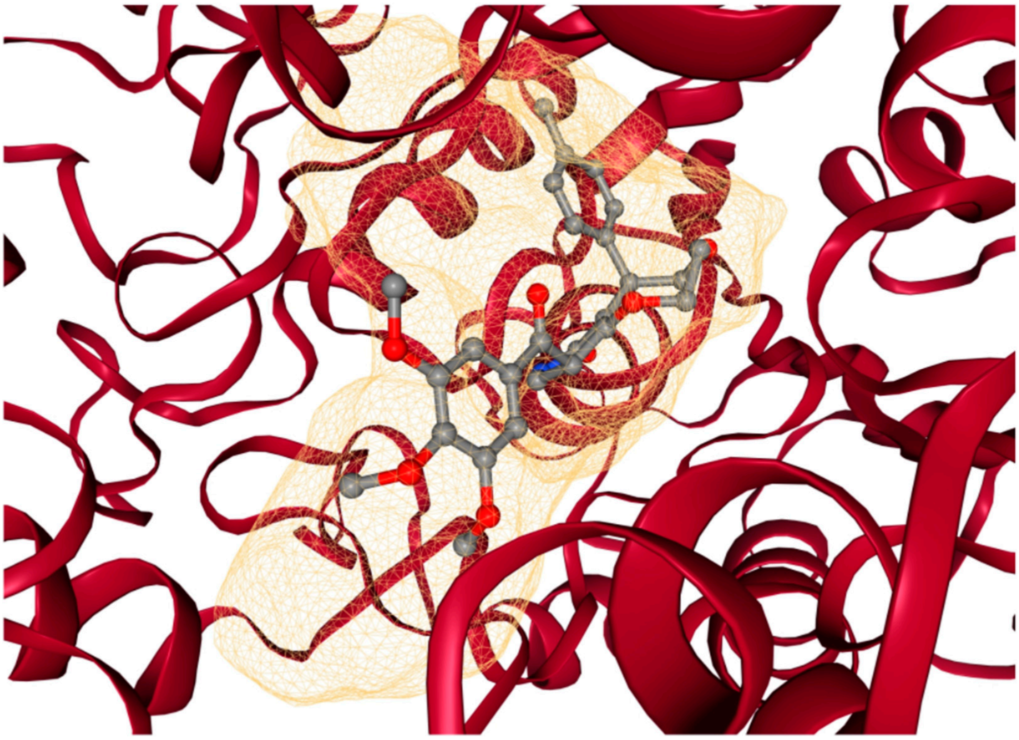

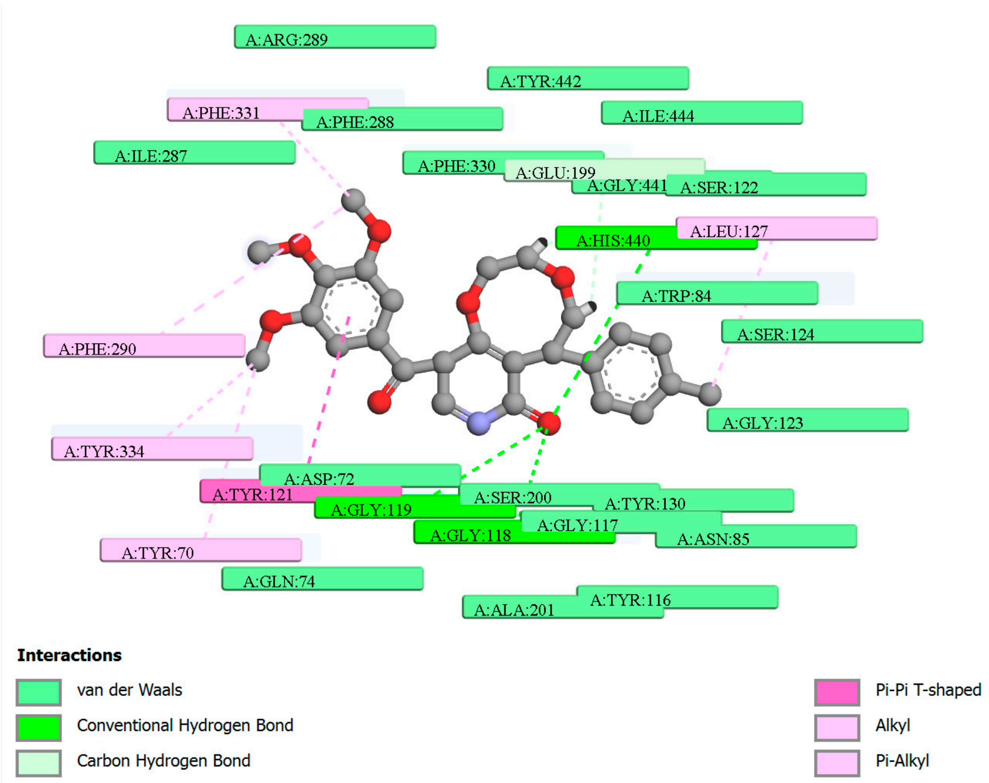
A:ARG:289
A:TYR:442
A:PHE:331
A:PHE:288
A:ILE:444
A:ILE:287
A:PHE:330
A:GLU:199
A:GLY:441
A:SER:122
A:HIS:440
A:LEU:127
A:TRP:84
A:SER:124
A:PHE:290
A:GLY:123
A:TYR:334
A:ASP:72
A:TYR:121
A:SER:200
A:TYR:130
A:GLY:119
A:GLY:117
A:GLY:118
A:ASN:85
A:TYR:70
A:GLN:74
A:TYR:116
A:ALA:201
Interactions
van der Waals
Conventional Hydrogen Bond
Carbon Hydrogen Bond
Pi-Pi T-shaped
Alkyl
Pi-Alkyl

Compound 15 – Tubulin

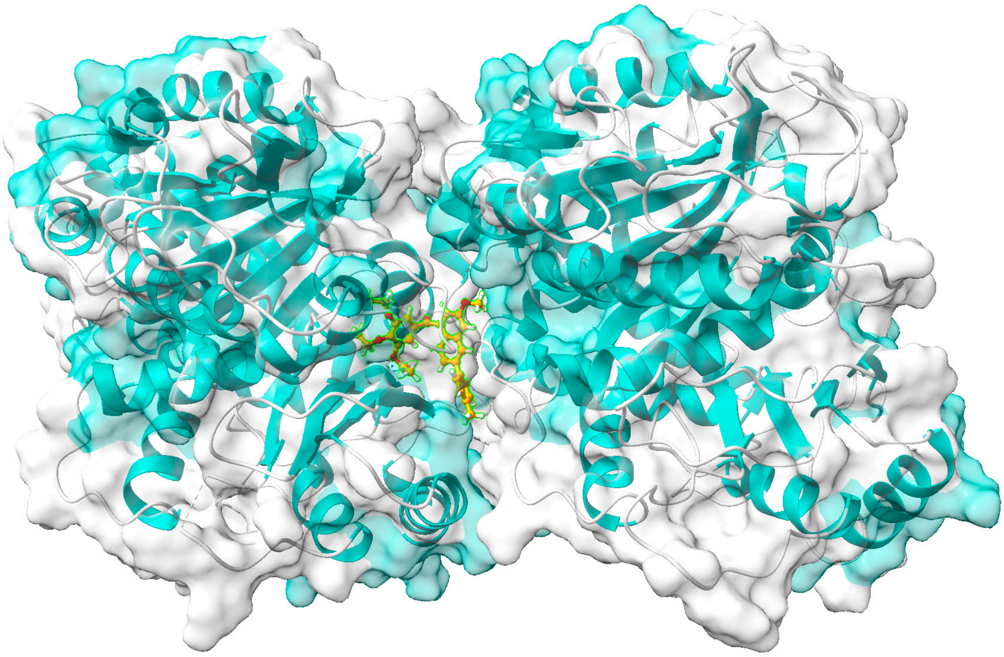

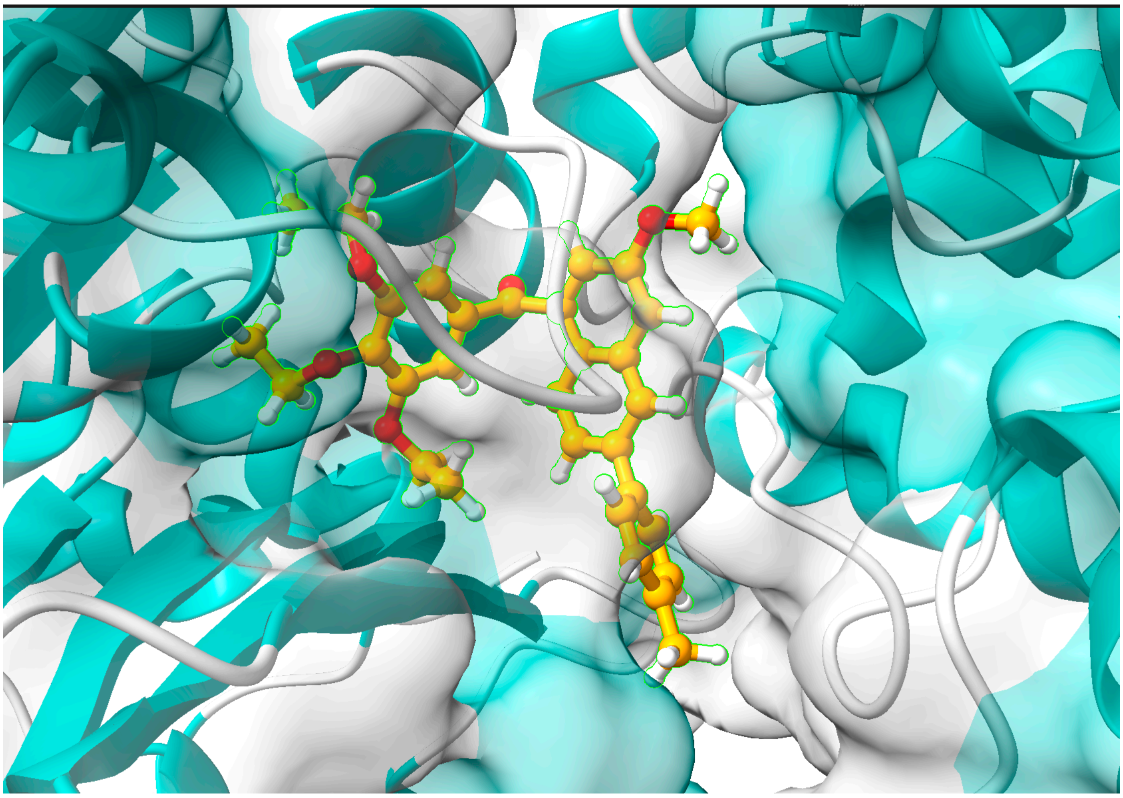

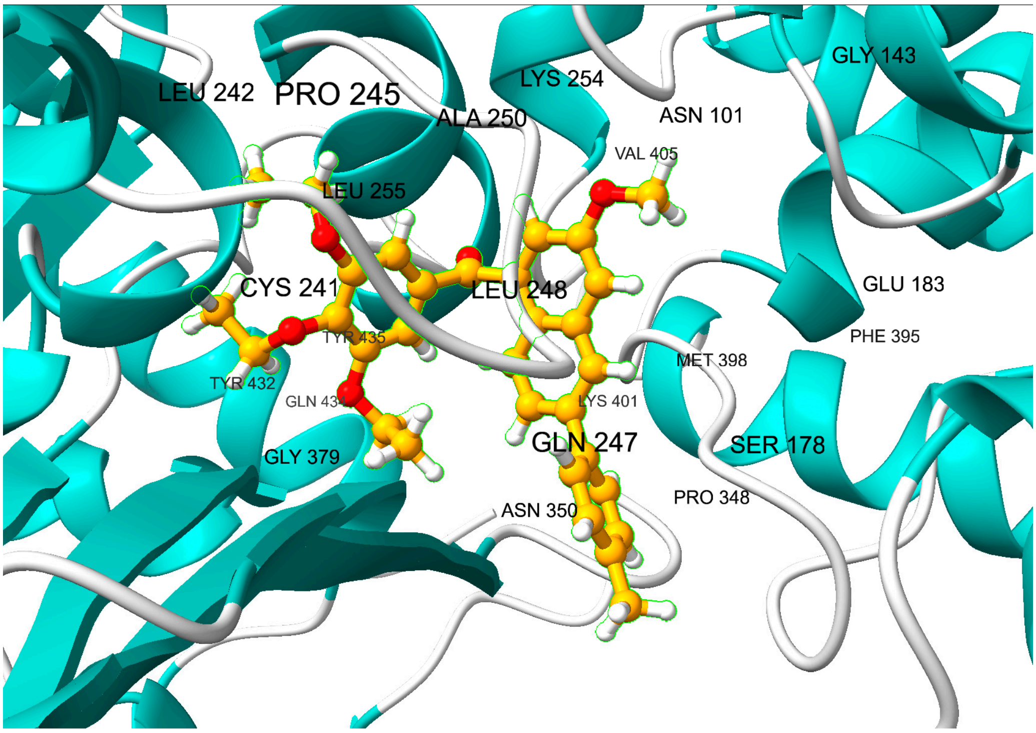
LEU 242
PRO 245
ALA 250
LYS 254
ASN 101
GLY 143
VAL 405
LEU 255
CYS 241
LEU 248
GLU 183
TYR 435
PHE 395
MET 398
TYR 432
LYS 401
GLN 247
SER 178
GLY 379
PRO 348
ASN 350

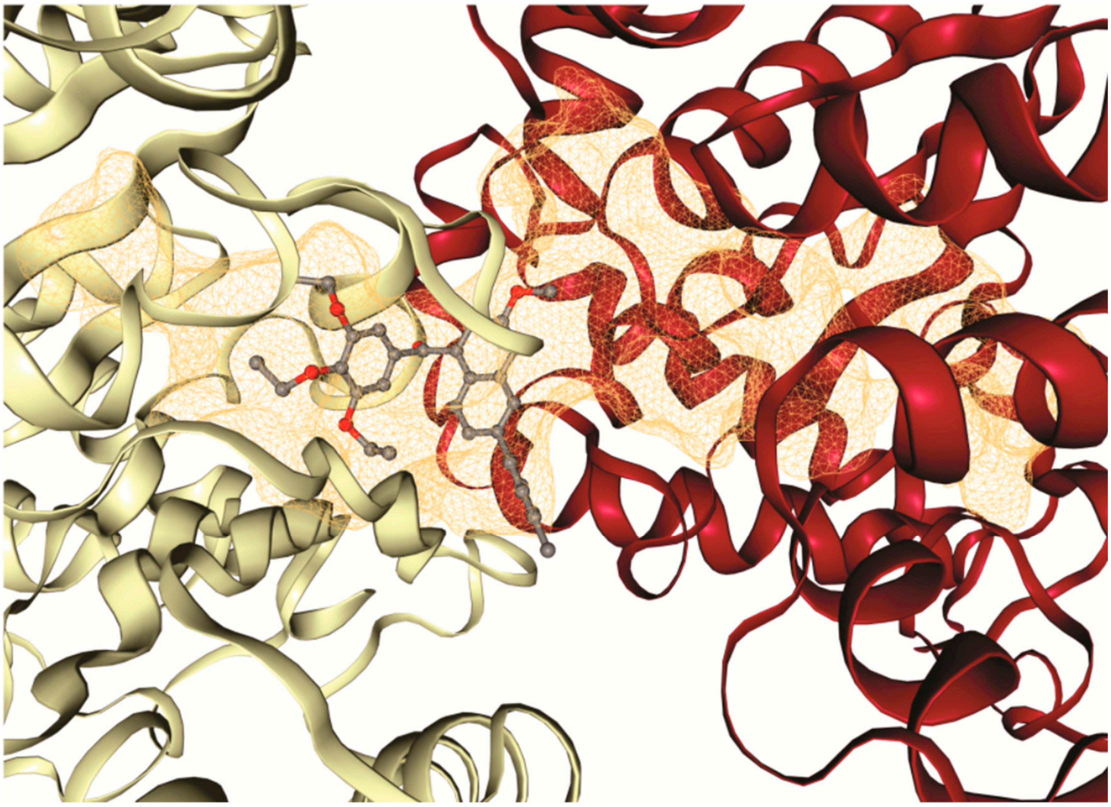

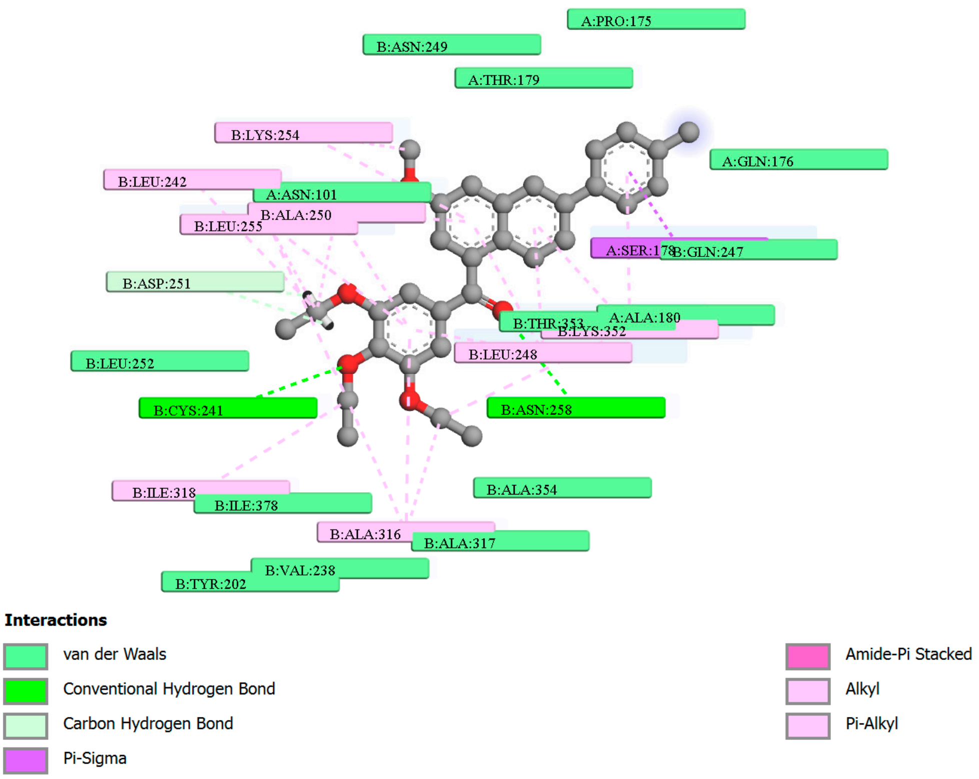
A:PRO:175
B:ASN:249
A:THR:179
B:LYS:254
A:GLN:176
B:LEU:242
A:ASN:101
B:ALA:250
B:LEU:255
A:SER:178
B:GLN:247
B:ASP:251
B:THR:353
A:ALA:180
B:LYS:352
B:LEU:252
B:LEU:248
B:CYS:241
B:ASN:258
B:ALA:354
B:ILE:318
B:ILE:378
B:ALA:316
B:ALA:317
B:VAL:238
B:TYR:202
Interactions
van der Waals
Conventional Hydrogen Bond
Carbon Hydrogen Bond
Pi-Sigma
Amide-Pi Stacked
Alkyl
Pi-Alkyl

Compound 16 – Tubulin

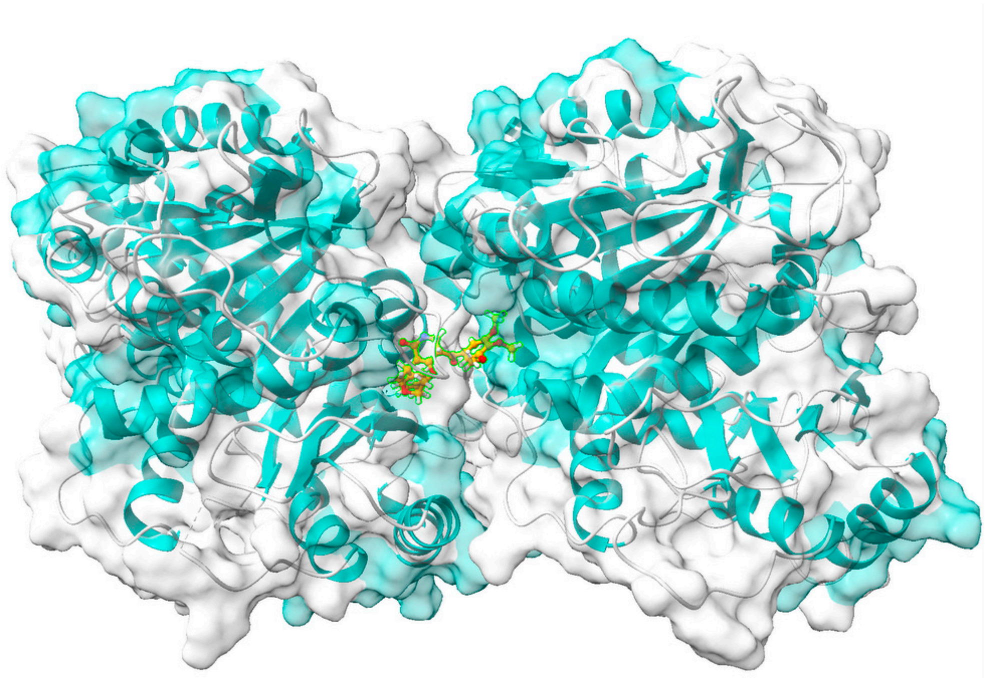

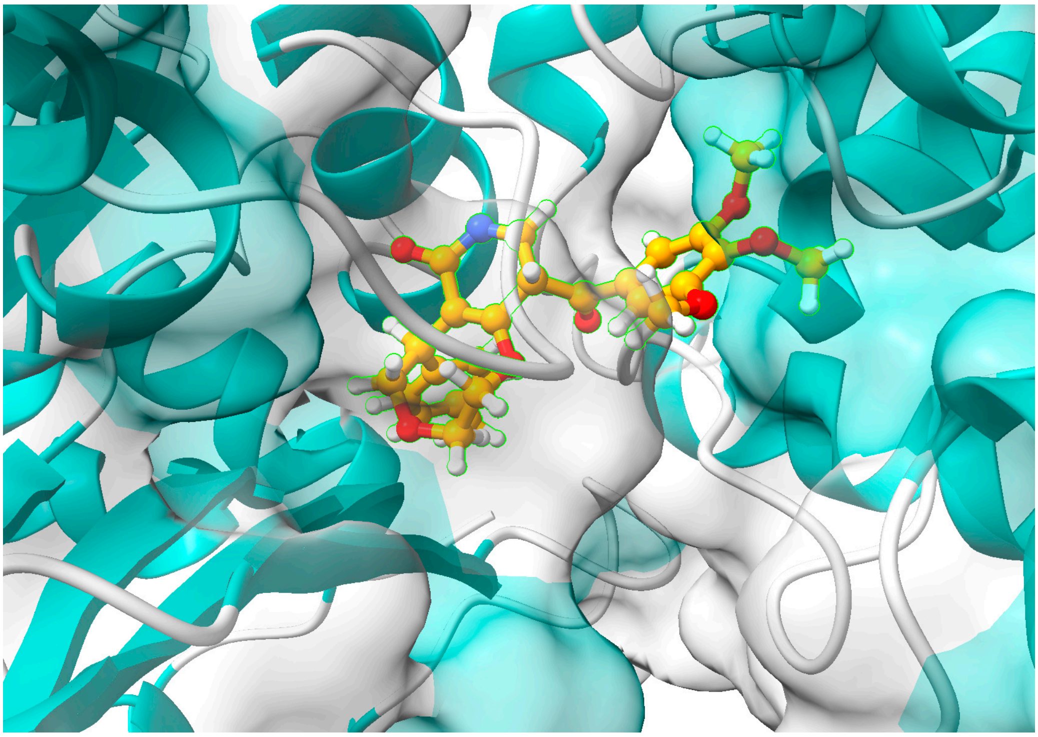

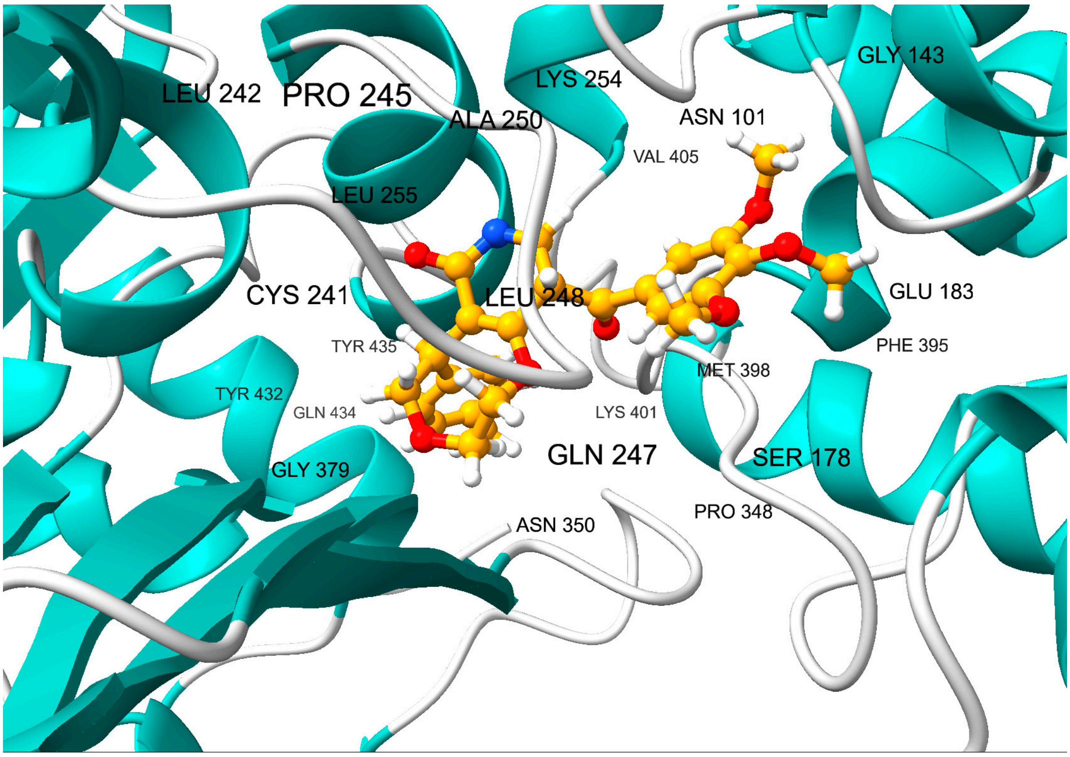
LEU 242
PRO 245
ALA 250
LYS 254
GLY 143
ASN 101
VAL 405
LEU 255
CYS 241
LEU 248
GLU 183
TYR 435
PHE 395
MET 398
TYR 432
GLN 434
LYS 401
GLN 247
SER 178
GLY 379
PRO 348
ASN 350

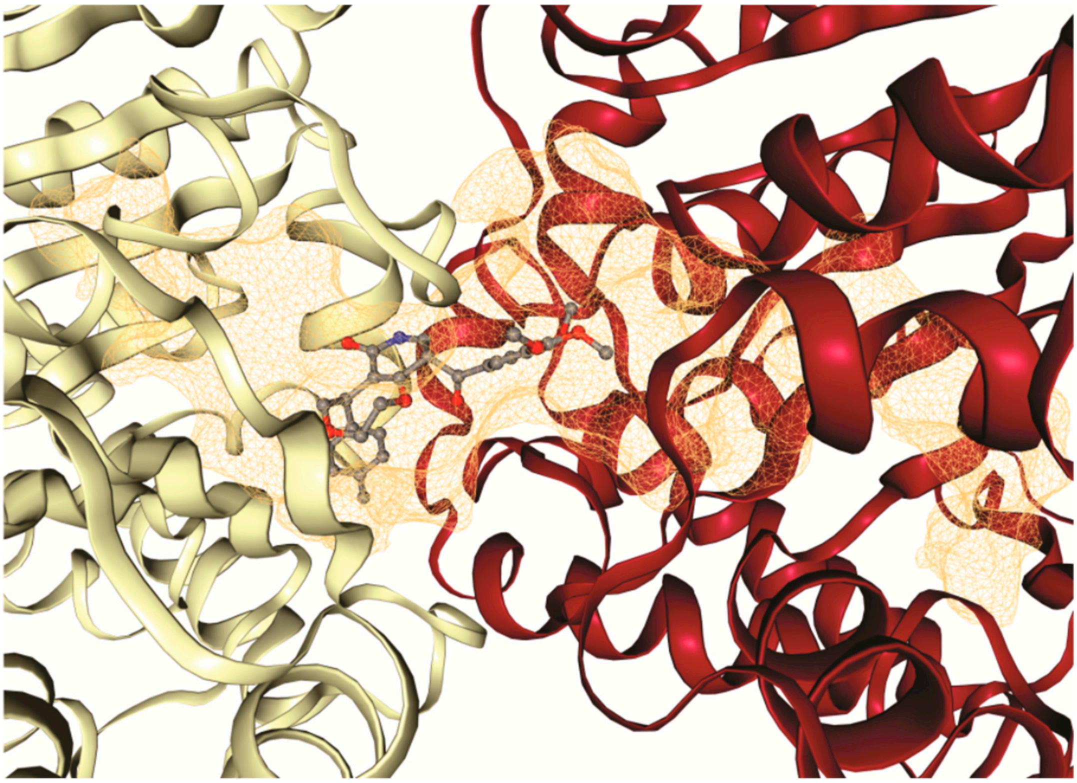

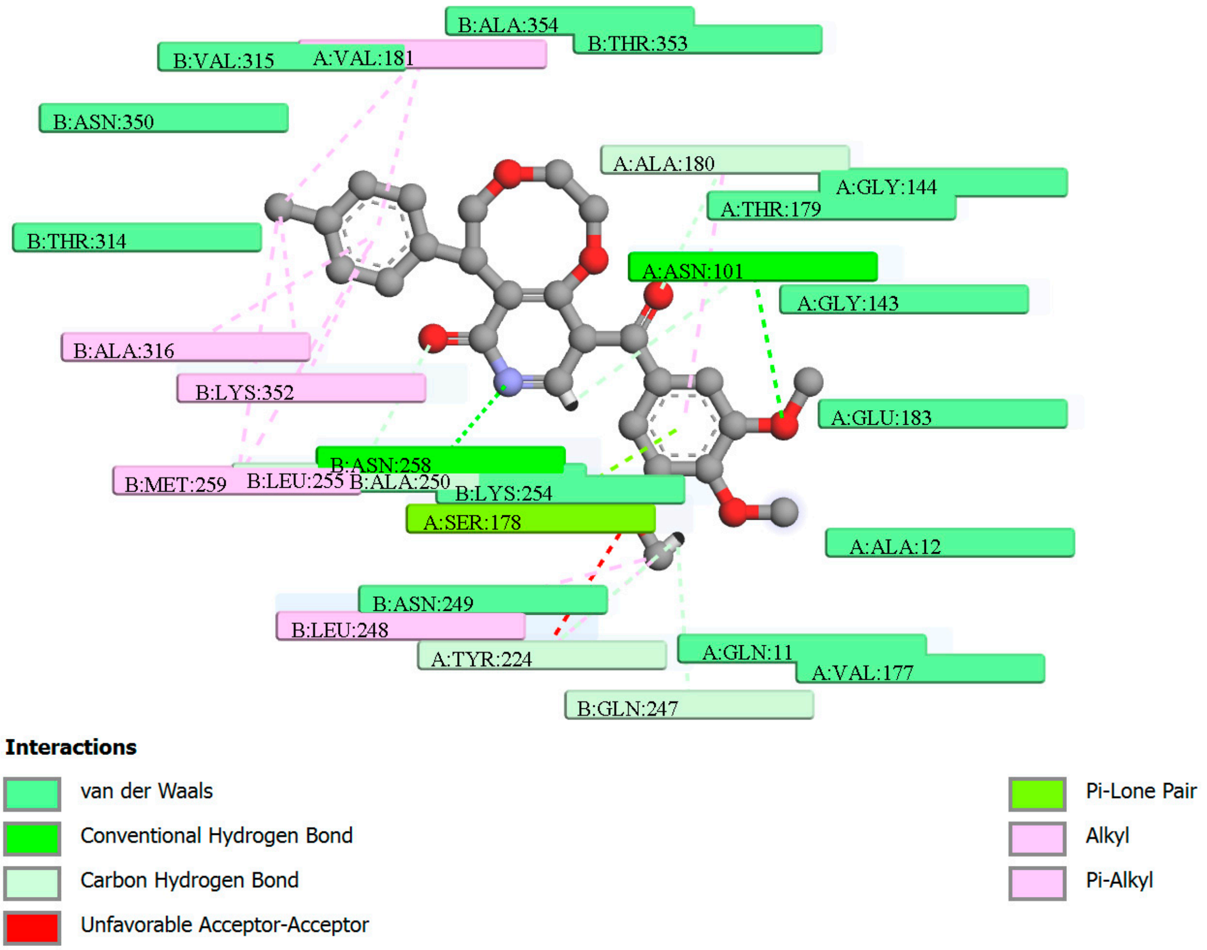
B:ALA:354
B:THR:353
B:VAL:315
A:VAL:181
B:ASN:350
A:ALA:180
A:GLY:144
A:THR:179
B:THR:314
A:ASN:101
A:GLY:143
B:ALA:316
B:LYS:352
A:GLU:183
B:ASN:258
B:MET:259
B:LEU:255
B:ALA:250
B:LYS:254
A:SER:178
A:ALA:12
B:ASN:249
B:LEU:248
A:TYR:224
A:GLN:11
A:VAL:177
B:GLN:247
Interactions
van der Waals
Conventional Hydrogen Bond
Carbon Hydrogen Bond
Unfavorable Acceptor-Acceptor
Pi-Lone Pair
Alkyl
Pi-Alkyl

S5. Molecular Dynamics (MD) Analysis Details

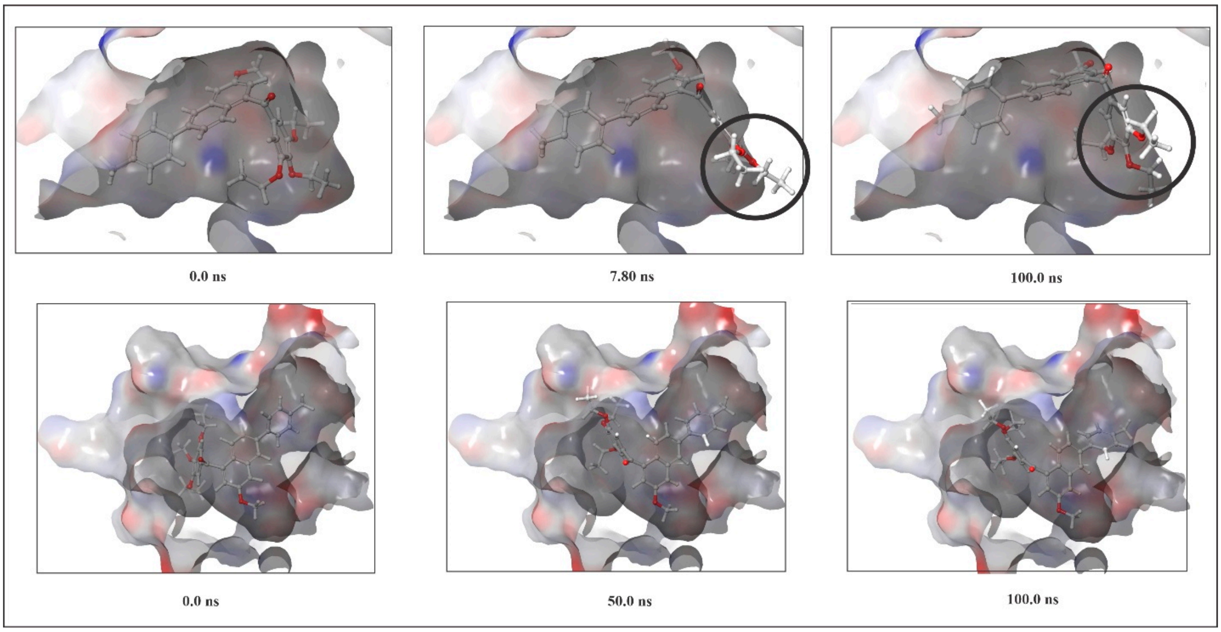

Figure S6a: Ligand fit during simulation with respect to primary docked position for **15.** (Top: AChE, Bottom: Tubulin)

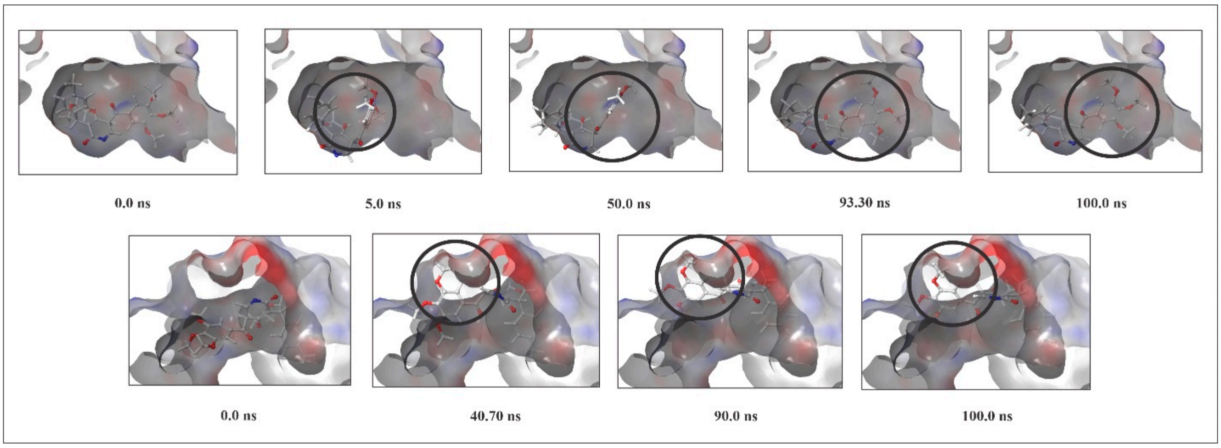

Figure S6b: Ligand fit during simulation with respect to primary docked position for **16.** (Top: AChE, Bottom: Tubulin)

S6. Ramchandran Plot Analysis:

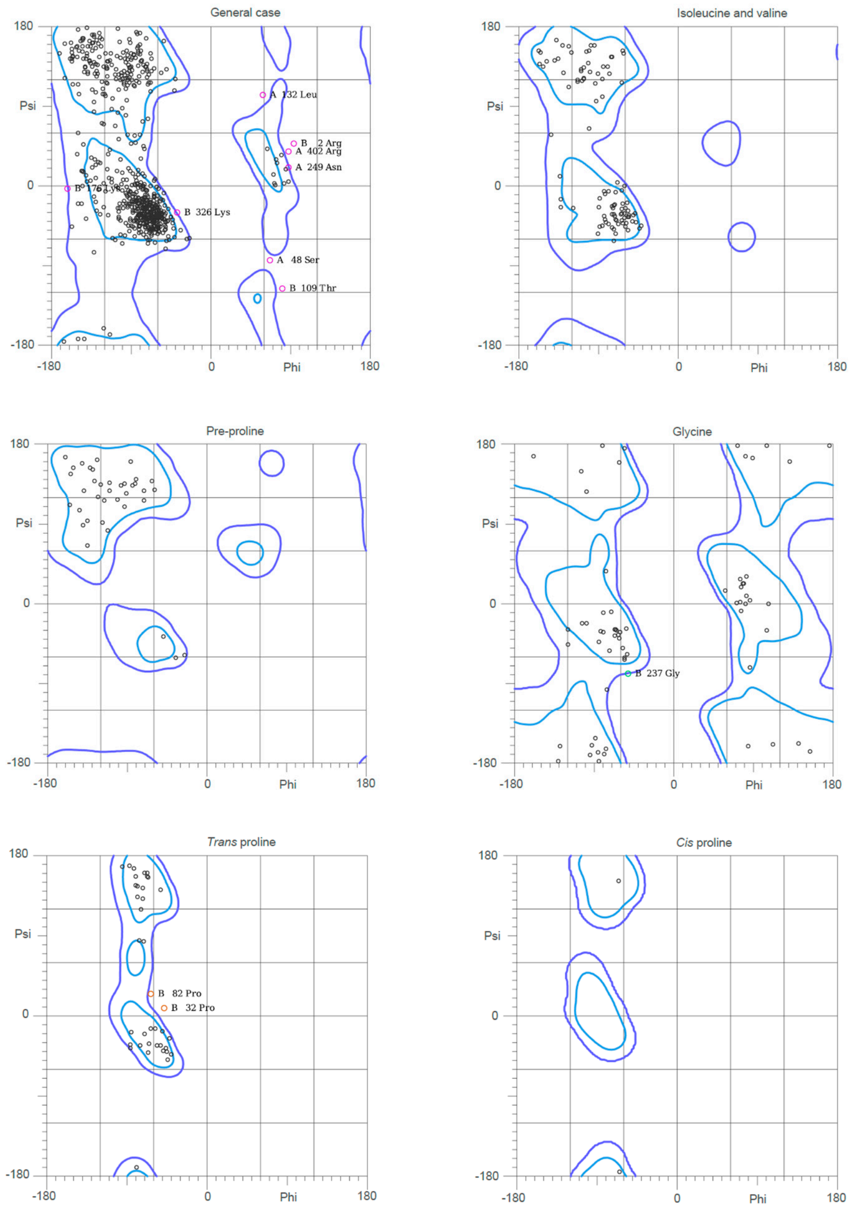

89.4% (764/855) of all residues were in favored (98%) regions.
98.7% (844/855) of all residues were in allowed (>99.8%) regions.

There were 11 outliers (phi, psi):

- A 48 Ser (68.0, -84.4)
- A 132 Leu (60.0, 103.7)
- A 249 Asn (88.6, 21.9)
- A 402 Arg (88.5, 39.6)
- B 2 Arg (94.7, 48.1)
- B 32 Pro (-48.6, 9.6)
- B 82 Pro (-63.8, 25.7)
- B 109 Thr (81.3, -116.5)
- B 176 Lys (-162.8, -3.4)
- B 237 Gly (-52.7, -79.1)
- B 326 Lys (-38.7, -30.7)

Figure S7a: Ramchandran analysis of Compound **15**-Tubuline

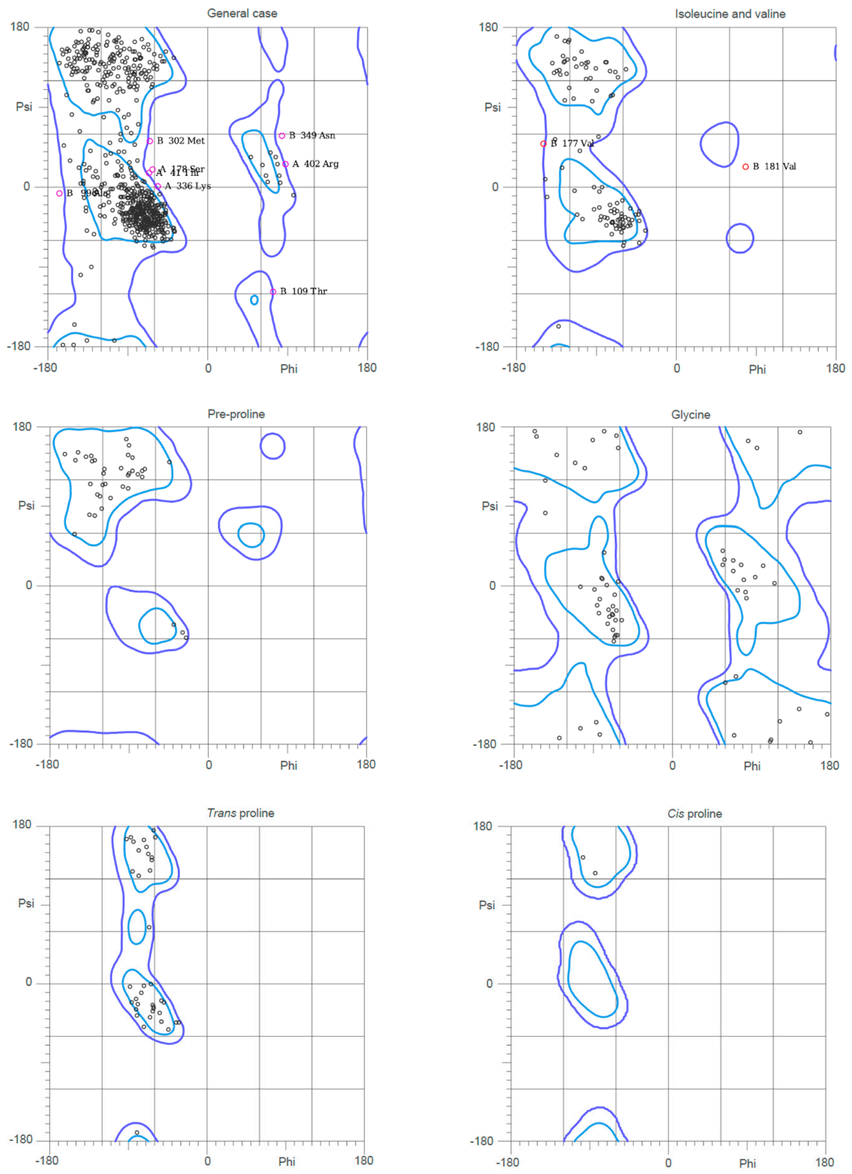

89.7% (767/855) of all residues were in favored (98%) regions.
98.8% (845/855) of all residues were in allowed (>99.8%) regions.

There were 10 outliers (phi, psi):

A 41 Thr (-66.8, 17.0)	B 302 Met (-65.3, 52.2)
A 178 Ser (-62.8, 20.3)	B 349 Asn (84.9, 58.9)
A 336 Lys (-56.6, 1.1)	
A 402 Arg (88.9, 26.9)	
B 99 Ala (-167.2, -7.8)	
B 109 Thr (74.2, -118.4)	
B 177 Val (-150.2, 49.5)	
B 181 Val (78.9, 23.1)	

Figure S7b: Ramchandran analysis of Compound **16**-Tubuline

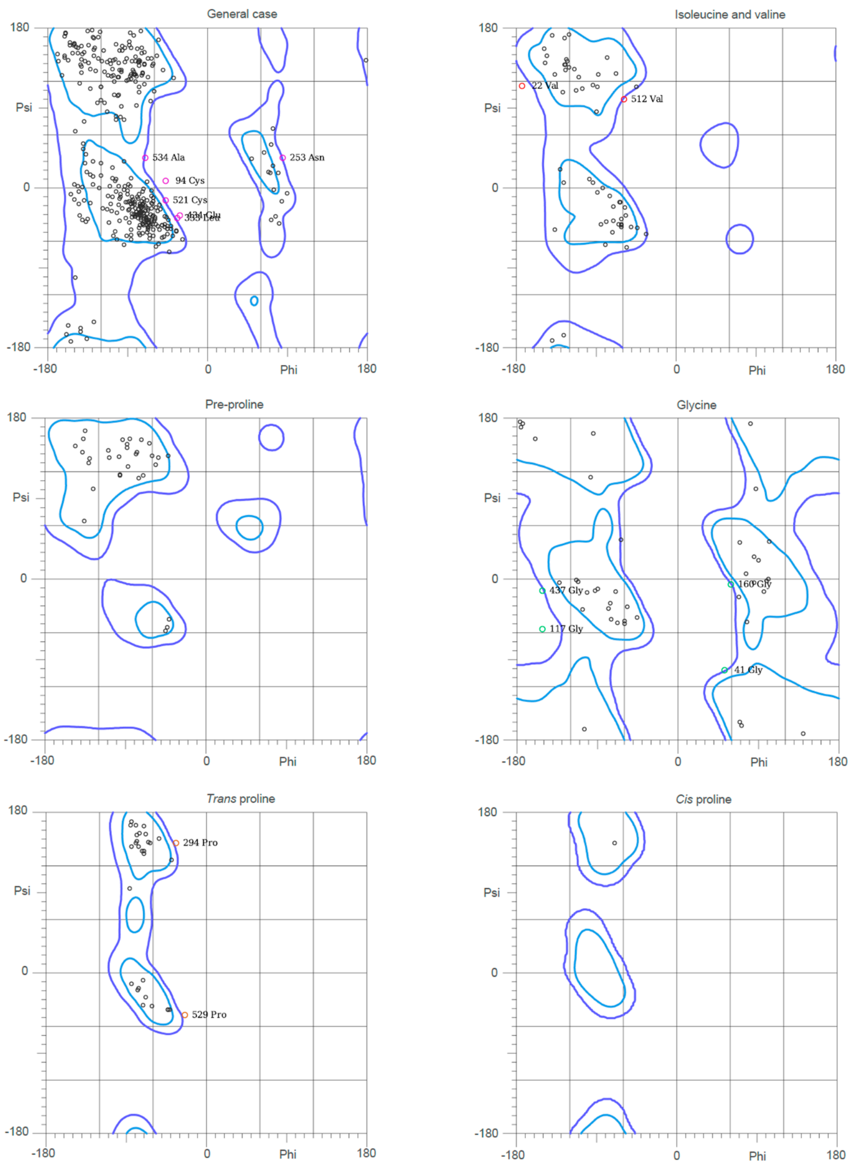

85.7% (456/532) of all residues were in favored (98%) regions.
97.4% (518/532) of all residues were in allowed (>99.8%) regions.

There were 14 outliers (phi, psi):

22 Val (-174.5, 115.3)	434 Glu (-31.8, -31.5)
41 Gly (53.0, -102.1)	437 Gly (-152.3, -13.4)
94 Cys (-47.8, 8.3)	512 Val (-59.0, 100.9)
117 Gly (-152.3, -56.3)	521 Cys (-47.1, -14.8)
160 Gly (59.1, -6.2)	529 Pro (-24.6, -47.7)
253 Asn (85.9, 34.8)	534 Ala (-70.1, 34.7)
294 Pro (-34.1, 147.0)	
333 Leu (-35.0, -34.7)	

Figure S7c: Ramchandran analysis of Compound **15**-AChE

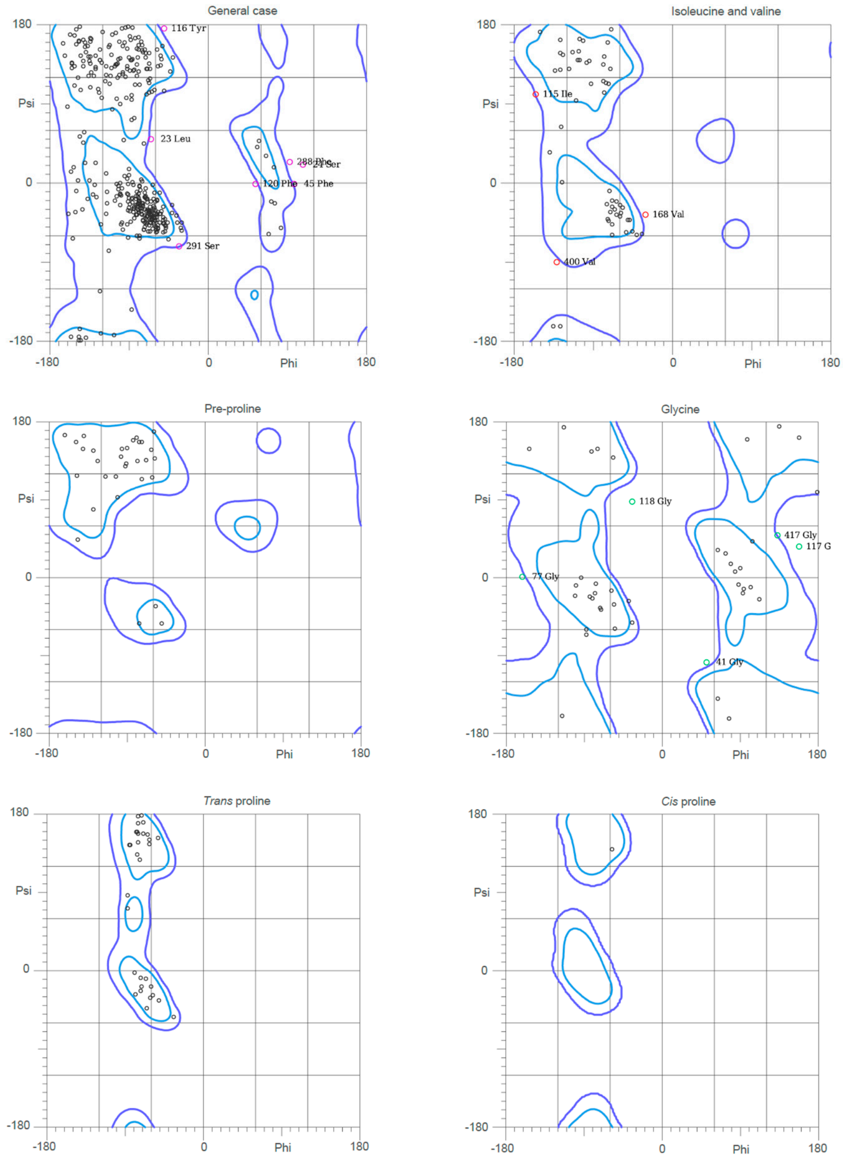

85.5% (455/532) of all residues were in favored (98%) regions.
97.2% (517/532) of all residues were in allowed (>99.8%) regions.

There were 15 outliers (phi, psi):

23 Leu (-65.4, 50.6)	118 Gly (-35.9, 88.4)
24 Ser (108.1, 21.1)	120 Phe (54.1, -1.3)
41 Gly (51.9, -98.5)	168 Val (-31.0, -37.0)
45 Phe (98.4, -1.6)	288 Phe (93.3, 24.2)
77 Gly (-162.3, 1.3)	291 Ser (-33.1, -72.6)
115 Ile (-156.9, 101.2)	400 Val (-132.8, -90.4)
116 Tyr (-50.1, 176.4)	417 Gly (133.6, 49.8)
117 Gly (158.6, 36.9)	

Figure S7d: Ramchandran analysis of Compound **16**-AChE

S7. DCCM Maps with Residue Labels:

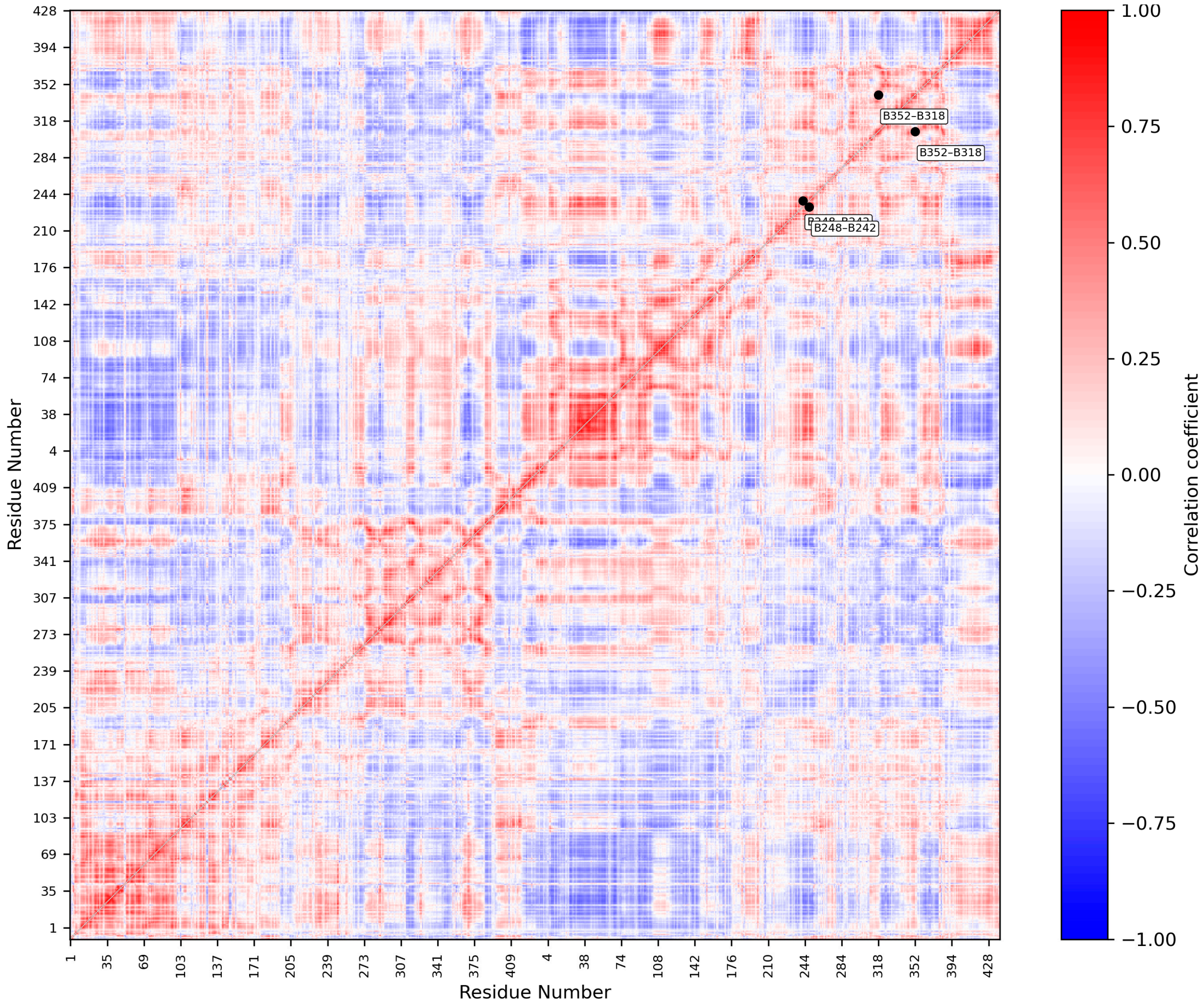

Figure S8a: DCCM Maps with Residue Labels of Compound **15**-Tubulin

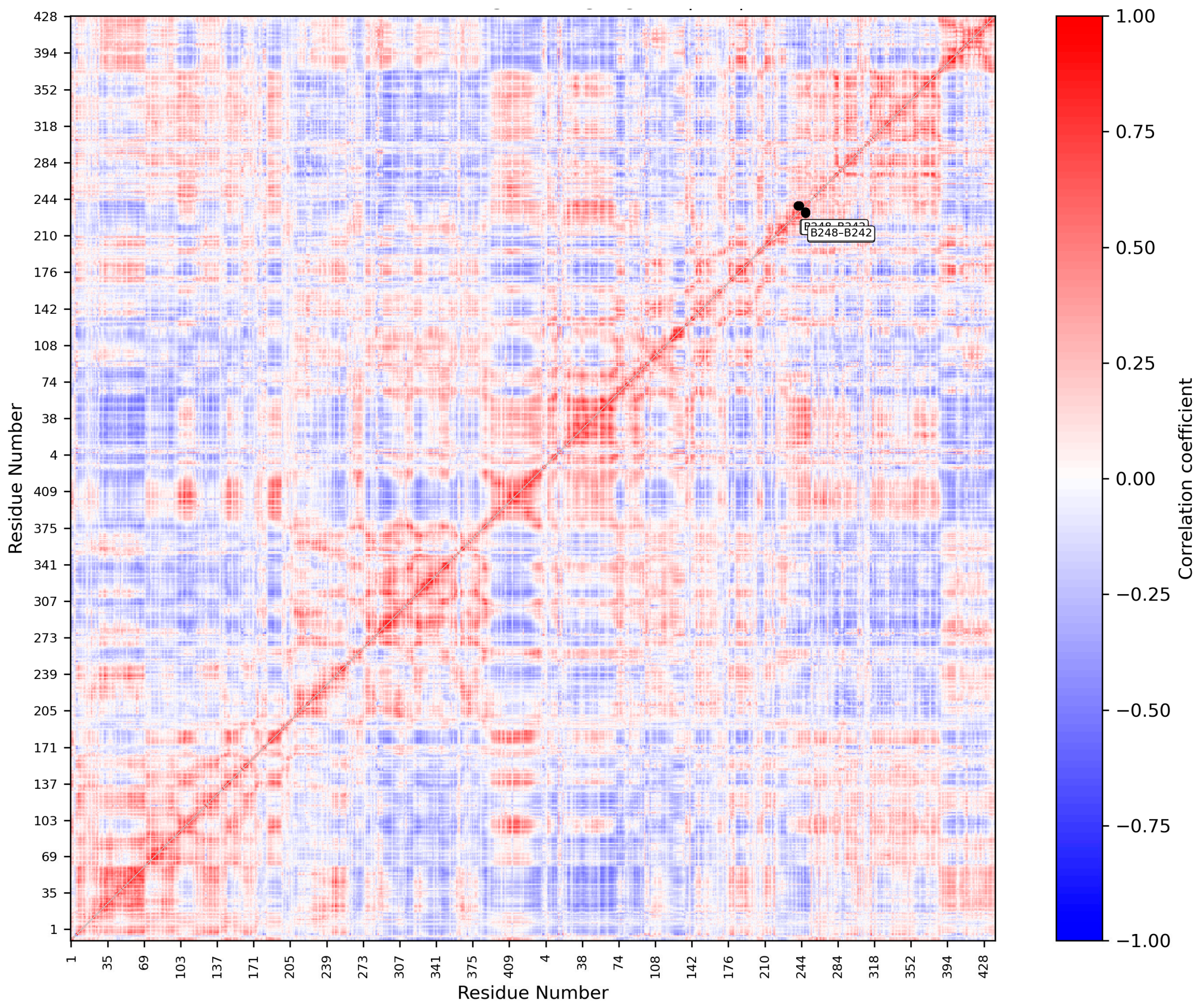

Figure S8b: DCCM Maps with Residue Labels of Compound **16**-Tubulin

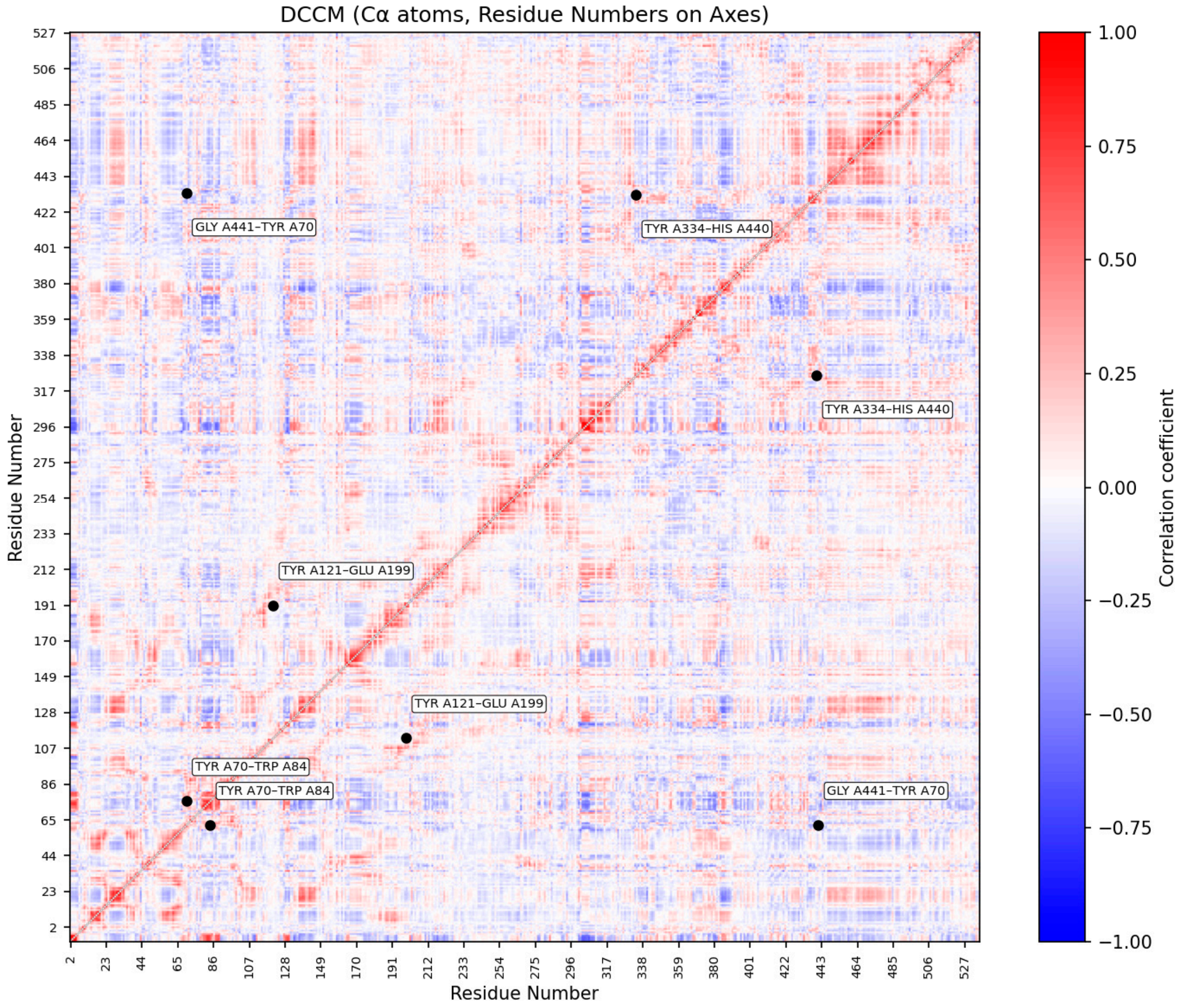

Figure S8c: DCCM Maps with Residue Labels of Compound **15**-AChR.

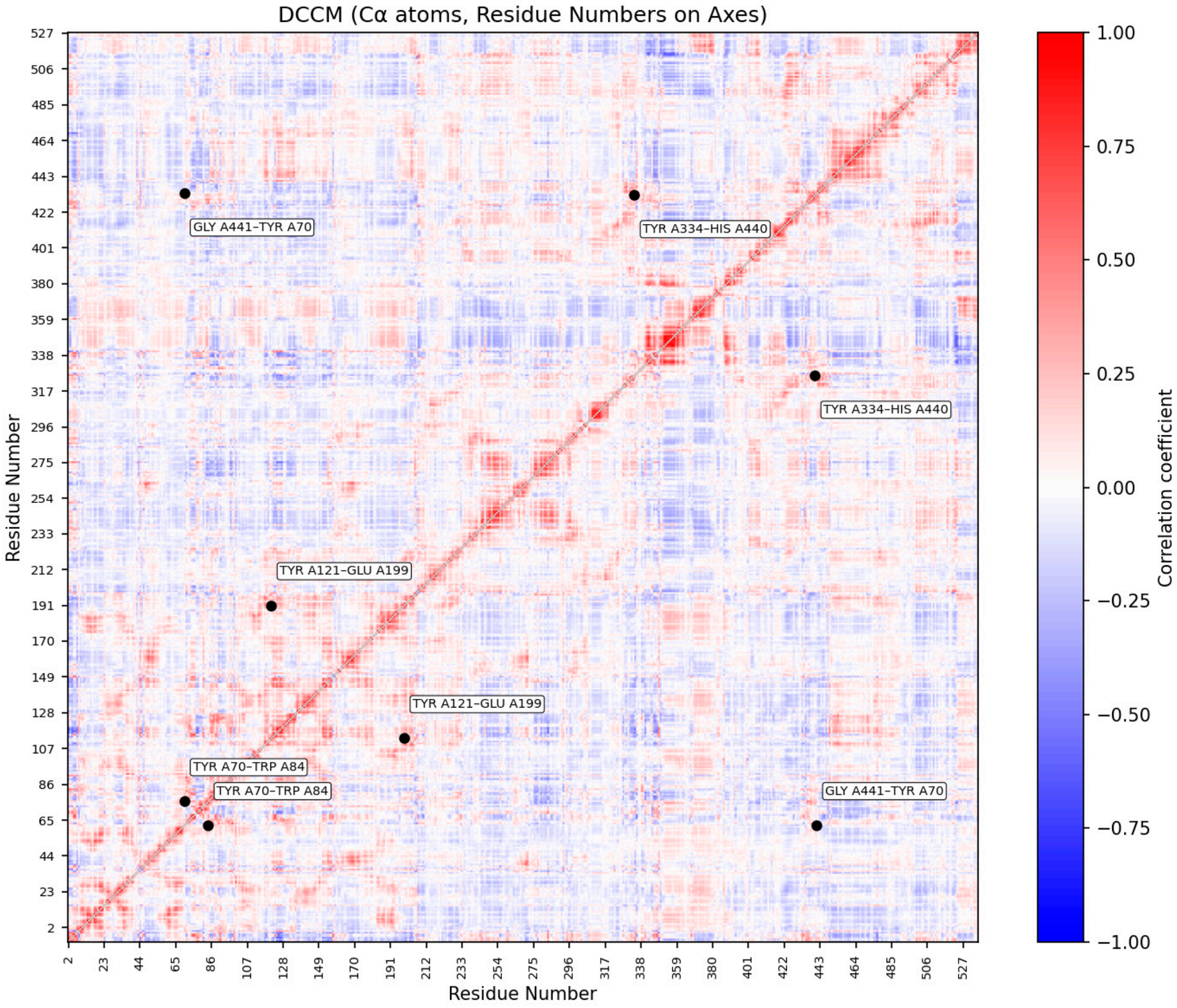

Figure S8d: DCCM Maps with Residue Labels of Compound **16**-AChR.
